# A Randomized, Placebo-Controlled Study of SRT2104, a SIRT1 Activator, in Patients with Moderate to Severe Psoriasis

**DOI:** 10.1371/journal.pone.0142081

**Published:** 2015-11-10

**Authors:** James G. Krueger, Mayte Suárez-Fariñas, Inna Cueto, Artemis Khacherian, Robert Matheson, Lawrence C. Parish, Craig Leonardi, Denise Shortino, Akanksha Gupta, Jonathan Haddad, George P. Vlasuk, Eric W. Jacobson

**Affiliations:** 1 Laboratory for Investigative Dermatology, The Rockefeller University, New York, New York, United States of America; 2 Center for Clinical and Translational Science, The Rockefeller University, New York, New York, United States of America; 3 Oregon Medical Research Center, PC, Portland, Oregon, United States of America; 4 Paddington Testing Co, Inc., Philadelphia, Pennsylvania, United States of America; 5 Central Dermatology, St. Louis, Missouri, United States of America; 6 Clinical Statistics, GlaxoSmithKline, Research Triangle Park, North Carolina, United States of America; 7 Sirtris, a GlaxoSmithKline Company, Cambridge, Massachusetts, United States of America; Kinki University Faculty of Medicine, JAPAN

## Abstract

Activation of Sirtuin (silent mating type information regulation 2 homolog) 1, or SIRT1, is an unexplored therapeutic approach for treatment of inflammatory diseases. We randomized 40 patients with moderate-to-severe psoriasis (4:1) to three escalating doses of SRT2104, a selective activator of SIRT1, or placebo. Across all SRT2104 groups, 35% of patients (p<0.0001) achieved good to excellent histological improvement based on skin biopsies taken at baseline and day 84 but was not consistently in agreement with PASI. Improvement in histology was associated with modulation of IL-17 and TNF-α signaling pathways and keratinocyte differentiation target genes. 27 subjects (69%) across all treatment groups, including placebo, experienced at least one treatment emergent adverse event. The majority of AEs were either mild or moderate. Most common were headache (8%), dizziness (8%), upper respiratory tract infection (8%), and psoriatic arthropathy (8%). Average drug exposure increased in a dose-dependent manner for escalating doses of SRT2104 and had high intra-subject variability in exposure (AUC %CV: 51–89%). Given the interesting signals of clinical activity, impact on gene expression and the generally favorable safety profile seen in this study, further investigation of SIRT1 activators for the treatment of psoriasis is warranted.

**Trial Registration:**

Clinicaltrials.gov NCT01154101

## Introduction

A novel therapeutic approach to treating psoriasis and other inflammatory diseases has emerged from research on calorie restriction (CR). Studies suggest that CR extends lifespan in lower organisms and mammals and improves a number of metabolic and inflammatory parameters. Sirtuin-1 (SIRT1), a NAD+ dependent class III histone deacetylase, has a number of cellular substrates including PGC-1α, NCoR, p300, NFκB, FOXO and p53 and has been implicated in regulation of metabolism, chronic inflammatory diseases, cancer and aging A direct role of SIRT1 in promoting keratinocyte differentiation has been shown and is supportive of earlier findings that resveratrol, a plant derived polyphenol which activates SIRT1, inhibited proliferation of human keratinocytes and suppressed angiogenesis *in vitro*. SRT2104 is novel, selective small molecule activator of SIRT1 that has been advanced into clinical development [1–4]. Pre-clinically, SRT2104 has shown efficacy following oral administration (10–300 mg/kg/day; for five to 28 days) in a number of *in vivo* inflammation models including lipopolysaccharide-induced TNF-α production [4]. Taken together with the finding that SIRT1 activators are effective in attenuating cytokine production, SIRT1 activation offers a potential new approach for treating psoriasis. In the present work, we hypothesized that SRT2104 as a selective SIRT1 activator may demonstrate anti-psoriatic activity by promoting keratinocyte differentiation, reducing inflammation and/or inhibiting angiogenesis.

## Materials and Methods

The protocol for this trial and supporting CONSORT checklist are available as supporting information; see S1 CONSORT Checklist and S1 Protocol.

### Ethical Conduct of the Study

This study was conducted in accordance with the ethical principles of the Declaration of Helsinki (version October, 2008) and the relevant regulations under 21 CFR parts 312, 50 and 56. A signed informed consent was obtained from each patient prior to performing any study related procedures. The study protocol and ICF were approved by institutional review boards of each participating study site (The Rockefeller University IRB, Schulman Associates IRB, and New York University IRB). The first subject visited on June 7, 2010 and the last subject visit was November 9, 2011 (clinicaltrials.gov NCT01154101).

### Study Subjects

Men and women aged 18 to 80 having clinically confirmed, stable plaque-psoriasis (without documented flare within 30 days prior to the screening visit) for at least 6 months involving ≥ 10% of body surface area were eligible to participate. Additionally, subjects had to be able and willing to provide written informed consent and had to have a baseline Psoriasis Area Severity Index (PASI) of ≥ 10 and were candidates for systemic psoriasis therapy, in the opinion of the investigator. Patients were excluded if they had received biologic agents within 5 half-lives (or within 3 months if half-life unknown) prior to first dose of study drug, systemic non-biologic psoriasis therapy or psoralen and ultraviolet light A (PUVA) phototherapy within four weeks prior to the screening visit, or had topical psoriasis treatment or ultraviolet light B (UVB) phototherapy within two weeks prior to the screening visit.

### Study Design

**Fig 1:**
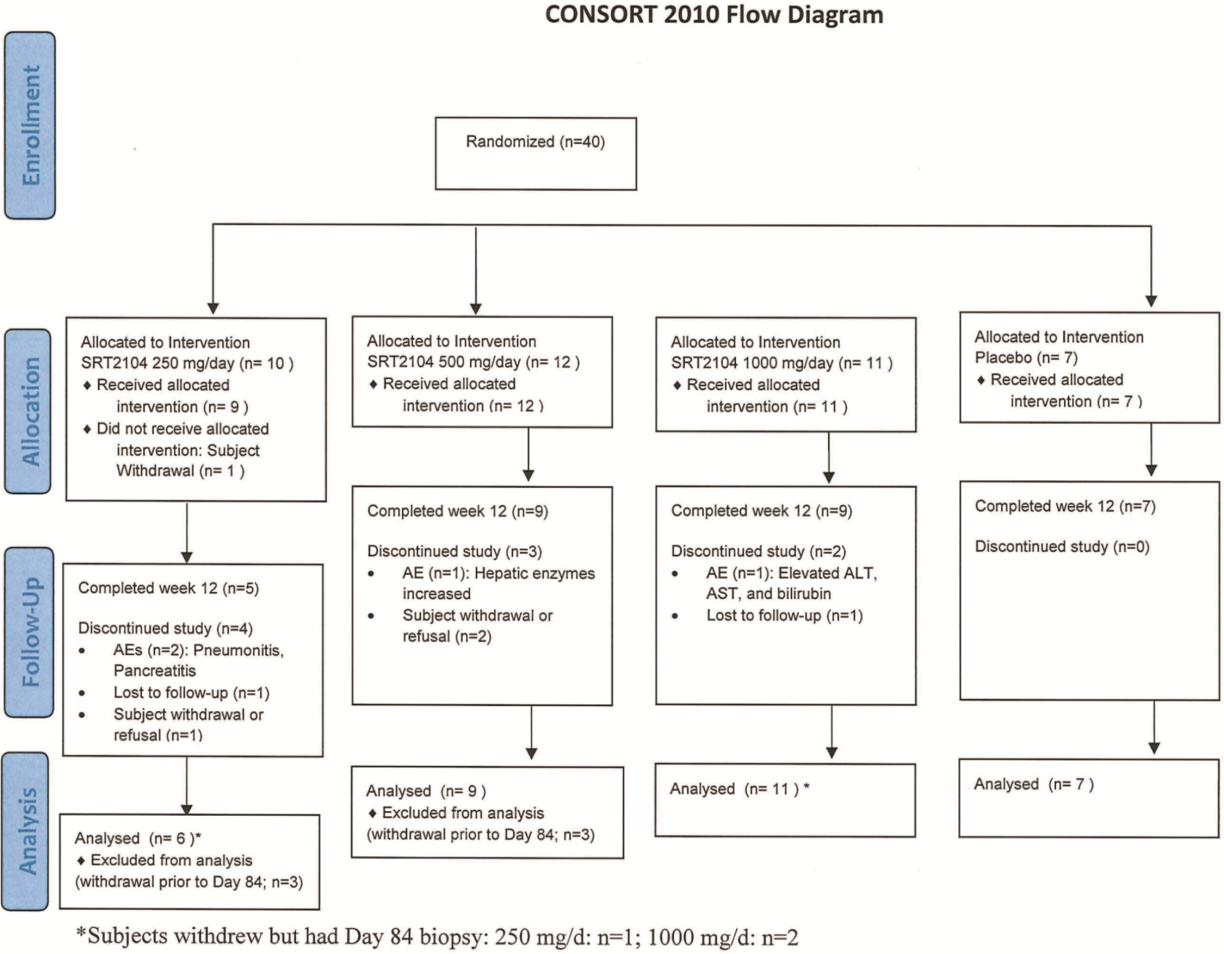
CONSORT 2010 Flow Diagram. The scheme graphically outlines the design and conduct of the clinical study.

The study was conducted at eight centers in the United States. This was a randomized, double-blind, placebo-controlled, Phase IIa, study with three dosing cohorts of approximately 10 subjects each (Fig 1). Subjects within each cohort were randomized 4:1, in a dose-escalating manner, to receive SRT2104 at one of the three doses– 250, 500 or 1000 mg / day or matching placebo for 84 consecutive days. Each cohort of subjects was dosed sequentially. Dosing in the second and the third cohort did not commence until subjects in the previous cohort completed at least 28 days of dosing and a review of safety parameters (physical examination findings, vital signs, ECGs, adverse events (AEs) and laboratory values) was completed by an Internal Safety Review Committee. All subjects were provided with one dosing bottle per day that contained one, two or four capsules (250 mg each, active or matched placebo) for oral ingestion. Subjects self-administered IP with 250 to 500 mL water, approximately 15 minutes following the consumption of a standardized meal, at the same time every dosing day.

No concomitant medications or herbal products were permitted during the trial except–i) medications administered to treat an AE, ii) topical class 6 and/or 7 rescue corticosteroid treatment for psoriasis flares to be applied only to face, axillae and groin (topical treatments were not to be applied to other areas, in particular the plaque being assessed for efficacy, at or near the biopsy site), and iii) chronically prescribed non-psoriatic medication or herbal or dietary supplements administered at a stable dose for at least 6 weeks prior to enrollment.

### Study Evaluations

The primary clinical activity endpoint was measured as a change in histological assessments of skin biopsies of psoriatic lesions from baseline to day 84 according to the following Histological Response Criteria:

- No improvement—No improvement in epidermal thickness, keratinocyte differentiation or K16 expression on keratinocytes
- Good improvement—Reduction in epidermal thickness by at least 30%, normalized keratinocyte differentiation, most keratinocytes still express K16
- Excellent improvement—Epidermal thickness reduced to normal or almost normal, normalized keratinocyte differentiation, absent keratinocyte expression of K16

Skin biopsies were obtained from the same designated plaque. A central blinded reader at Rockefeller University (Dr. J. Krueger) evaluated the biopsy tissue for general appearance, epidermal thickness, total inflammatory infiltrate and Keratinocyte expression of K16. The histological analysis followed the parameters for the study by Zaba et al [5]. Secondary clinical activity end points were the assessment of effect of SRT2104 on PASI and Physician Global Assessment (PGA) [6,7] scores in patients with moderate to severe plaque psoriasis after 28, 56, and 84 days of exposure. Additional objectives of the study included assessment of the pharmacodynamic effects of SRT2104 as measured by biomarkers of psoriasis and/or sirtuin pathway activation (including hsCRP, FGF21) and determination of pharmacokinetic (PK) profile. A sparse sampling approach was used to collect PK samples. Area under the Curve (AUC) of SRT2104 for a steady state dosing interval (0 to 24 hours) was computed using population PK modeling; Cmax was based on the highest observed plasma concentration. Pearson’s correlation was used to assess association between skin concentration and plasma concentration, and PASI response. Subjects were assessed for safety and tolerability by adverse events, laboratory parameters, physical examination, vital signs and 12-lead ECGs throughout the study.

### Microarray Analysis

Quality control of microarray chips was carried out using standard QC metrics and R package microarray Quality Control Images were scrutinized for spatial artifacts using Harshlight [8]. Expression measures were obtained using GCRMA algorithm [9]. Probe-sets with at least one sample with expression values larger than 4 and standard deviation (SD)>0.1 were kept for further analysis. Expression values were modeled using mixed-effect models with fixed factors Time and Group and a random effect for each patient using an unstructured covariance analysis. Comparisons of interests were tested using contrasts under the general framework for linear models in *limma* package. P-values from the moderated t-test resultant were adjusted for multiple hypotheses using the Benjamini–Hochberg procedure [10]. Genes showing fold change (FC) >2 and false discovery rate (FDR) <0.1 were considered to be differentially expressed genes (DEG). Hierarchical Clustering were obtained using Euclidean distance and mcquitty agglomeration scheme.

### Gene Expression

Skin biopsies were snap-frozen in liquid nitrogen and stored at −80°C until used. RNA was extracted using the Qiagen RNeasy Fibrous Tissue Mini Kit (QIAGEN, Valencia, CA) and later labeled and hybridized to GeneChip HG U133 Plus 2.0 (Affymetrix, Santa Clara, CA) using WT-Ovation Pico RNA Amplification system (Nugen). Raw data have been deposited in NCBI's Gene Expression Omnibus and are accessible through accession number GSE50614.

### Statistical Analysis

The primary clinical activity endpoint was the Histological Response, which was based on histological assessment of skin biopsies after day 84 of treatment (defined in the study evaluation section). Sample size was based on feasibility rather than statistical operating characteristics. The primary, prospectively defined analysis of clinical activity was a comparison of the proportion of subjects achieving good or excellent improvement relative to a historical placebo response rate of 5%. A one-sample, one-sided binomial test was used to assess statistical significance at the alpha 0.05 level. Normality assumptions were not tested. Because previous studies have indicated that the pharmacokinetic exposure is relatively variable [11], an exposure-response analysis was used to further evaluate activity. The imputed AUCs from the SRT2104 treated groups were natural-log transformed and dichotomized at the midpoint for a low and high exposure group. Point estimates and 90% exact confidence intervals (CI) were constructed for differences between the proportion of responses, defined as “good” or “excellent” histological improvement, for each of the SRT2104-treated groups (250mg, 500mg, and 1000mg), exposure groups (High and Low) and the placebo group. Given the small sample size, less stringent 90% CIs were considered appropriate. No adjustments were made for multiple comparisons and no missing values were imputed.

Point estimates and 90% exact confidence intervals based on the Blyth-Still-Casella method [12] were constructed for the secondary endpoints of clinical activity (PASI-50 (data not shown) and PGA of “minimal” or “not clear”) for the difference between the proportion of responders for each of the SRT2104-treated groups, exposure groups and the placebo group. Mixed effects ANOVA was used to estimate mean PASI over time; model terms included baseline PASI, treatment, day and treatment-by-day interaction. Descriptive statistics (mean, SD, min, max and 90% CI) of the percent change in PASI are provided for SRT2104-treated groups, exposure groups and placebo group. Post hoc exploratory analyses of response predictors were performed using a stepwise linear regression for Day 84 total PASI score and stepwise logistic regression for Day 84 PASI25, which was an indicator variable if the subject responded with at least a 25% reduction in PASI. Baseline PASI was entered in these models. Parameters were entered/retained in the model if p< = 0.2000.

## Results

### Patients

The CONSORT 2010 flow diagram of this study is shown in Fig 1. A total of forty patients were randomized to oral SRT2104 at 250 mg, 500 mg or 1000 mg/d SRT2104 or placebo for 84 days of treatment. Thirty out of 40 patients (75%) completed the treatment. One subject withdrew from the study prior to dosing. A total of 9 patients discontinued treatment–the reasons were AEs (n = 4), subject withdrawal or refusal (n = 3) and lost to follow-up (n = 2) (Fig 1). AEs leading to withdrawal included pancreatitis (n = 1), pneumonitis (n = 1), and LFT elevations (n = 2). All patients who received at least one dose of treatment (n = 39) were included in the safety analysis. All patients who received at least one dose of investigational product (IP), had at least one activity measurement at baseline, and at least one post-baseline study visit (n = 39) were included in the analysis of clinical activity. The majority of the study populations were Caucasian (85%) men (75%). The mean age was 46.4 years. The BMI was higher for the SRT2104 250 mg dose group (33.4 kg/m^2^) compared to the 500 mg and 1000 mg and placebo groups (27.4 kg/m^2^, 28.6 kg/m^2^ and 31.5 kg/m^2^, respectively (Table 1).

**Table 1:** Patient Demographics^1^.

	Placebo	SRT2104 Dose		
Parameter	(N = 7)			
		250 mg (N = 9)	500 mg (N = 12)	1000 mg (N = 11)
**Age (Years)**				
N	7	9	12	11
Mean (SD)	51.4 (14.9)	40.6 (16.1)	49.3 (14.4)	44.6 (13.6)
Median (Min, Max)	50 (27, 72)	45 (19, 60)	52 (26, 68)	43 (26, 65)
**Sex**, n (%)				
n	7	9	12	11
Women:	1 (14)	3 (33)	0	5 (45)
Men:	6 (86)	6 (67)	12 (100)	6 (55)
**Race,** n (%)				
n	7	9	12	11
White	6 (86)	7 (78)	10 (83)	10 (91)
Black	1 (14)	2 (22)	0	1 (9)
Asian	0	0	1 (8)	0
Other	0	0	1 (8)	0
**Ethnicity**, n (%)				
n	7	9	12	11
Hispanic or Latino	0	0	3 (25)	2 (18)
Not Hispanic or Latino	7 (100)	9 (100)	9 (75)	9 (82)
**Height (cm)**				
n	7	9	10	11
Mean (SD)	172 (7.99)	176 (6.86)	177 (7.81)	173 (7.72)
Median (Min, Max)	175 (155, 178)	178 (165, 185)	177 (160, 185)	175 (160, 188)
**Weight (kg)**				
n	7	9	12	11
Mean (SD)	92.7 (11.2)	105 (24.5)	88.1 (15.6)	84.9 (27.8)
Median (Min, Max)	91.9 (77, 104)	105 (74, 156)	81.1 (70, 116)	81.6 (62, 158)
**BMI (kg/m** ^**2**^ **)** ^1^				
n	7	9	10	11
Mean (SD)	31.5 (3.81)	33.4 (5.57)	27.4 (4.40)	28.6 (9.63)
Median (Min, Max)	32.9 (24, 36)	33.4 (26, 45)	25.9 (23, 35)	25.8 (20, 55)

^1^ BMI–body mass index.

### Efficacy

Across all active treatment groups, 35% (9 out of 26 subjects) achieved Good or Excellent Histological Improvement based on skin biopsy analysis when compared to the historical placebo rate of 5% (p<0.0001; 90% CI 18.0%, 54.2%) (Table 2). A higher proportion of subjects with good to excellent histological improvement were observed in SRT2104 250 mg (n = 3, 50.0%, 90% CI 15.3%, 84.7%) and 500 mg (n = 4, 44.4%, 90% CI 16.9%, 74.9%) groups as compared to placebo (n = 1, 14.3%, 90% CI 0.7%, 55.4%) and the 1000 mg group (n = 2, 18.2%, 90% CI 3.3%, 50.0%). A higher proportion of subjects in the low exposure group had histological improvement (41.7%) as compared to the high exposure group (28.6%).

**Table 2:** Proportion of Subjects with Good-Excellent Histological Improvement vs. Historical 5% Placebo rate–Day 84.

Comparison to 5% Historical Placebo Response	n	Good or Excellent Improvement, n (%)	90% CI	p-value
Placebo (N = 7)	7	1 (14.3)	(0.73, 55.4)	—
SRT2104 250 mg (N = 9)	6	3 (50)	(15.3, 84.7)	0.0022
SRT2104 500 mg (N = 12)	9	4 (44.4)	(16.9, 74.9)	0.0006
SRT2104 1000 mg (N = 11)	11	2 (18.2)	(3.33, 50.0)	0.1019
All Active (N = 32)	26	9 (34.6)	(18.0, 54.2)	<0.0001
Low Exposure (N = 15)	12	5 (41.7)	(18.1, 70.6)	0.0002
High Exposure (N = 16)	14	4 (28.6)	(10.4, 58.1)	0.0042

**Fig 2:**
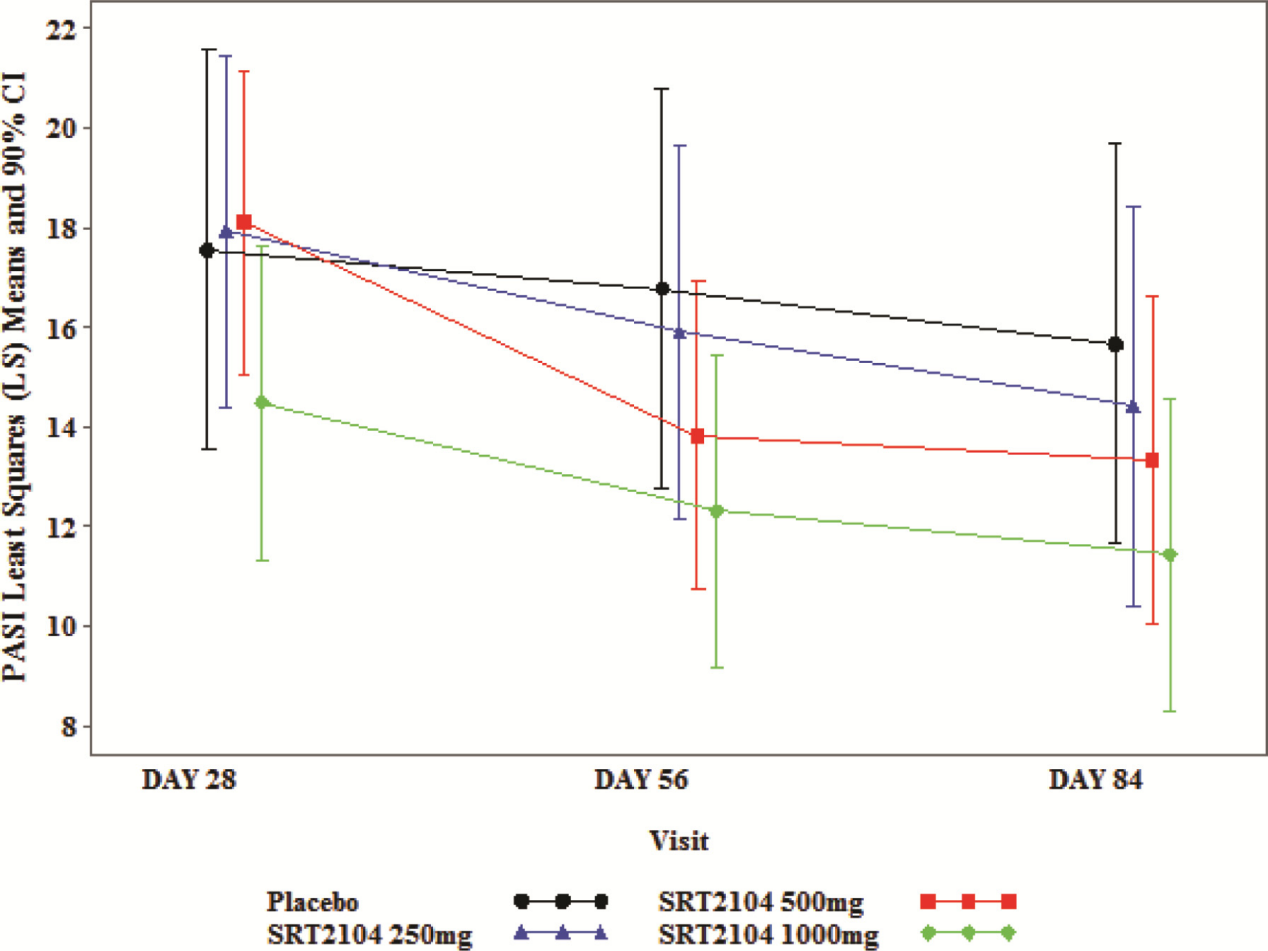
Plot of LS Mean (+/- SE) PASI Total Score vs. Time.

Improvement in adjusted mean PASI scores across the three active treatment groups was seen over time with an apparent dose/exposure-response. On day 84, adjusted mean PASI was 15.65 in the placebo group, vs. 14.41, 13.32, and 11.43 in the 250 mg, 500 mg and 1000 mg groups, respectively. When categorized by exposure groups, adjusted mean PASI scores on day 84 were lower for the higher exposure group than for the low exposure group (11.59 vs. 14.24, respectively) which was sustained through follow-up (Table 3, Fig 2). PGA improvements were greater for subjects receiving SRT2104 compared to those receiving placebo. On Day 84, no subjects in the placebo group achieved PGA scores of minimal/clear whereas two subjects (22%) in 500 mg group and three subjects (27%) in 1000 mg group achieved minimal/clear scores (all 5 of these responses were observed in the high exposure group (S2 Table). The 250 mg dose group was not evaluated due to an operational error.

**Table 3:** Adjusted Mean PASI Day 84.

Group (N)	Study Day	LS Mean for Treatment	LS Mean for Placebo	Estimated Difference	90% CI of Difference
SRT2104 250mg	84	14.41	15.65	-1.249	(-7.00, 4.51)
SRT2104 500mg	84	13.32	15.65	-2.336	(-7.58, 2.91)
SRT2104 1000mg	84	11.43	15.65	-4.225	(-9.30, 0.85)
Low Exposure	84	14.24	15.74	-1.501	(-6.53, 3.53)
High exposure	84	11.59	15.74	-4.151	(-9.02, 0.72)

**Fig 3:**
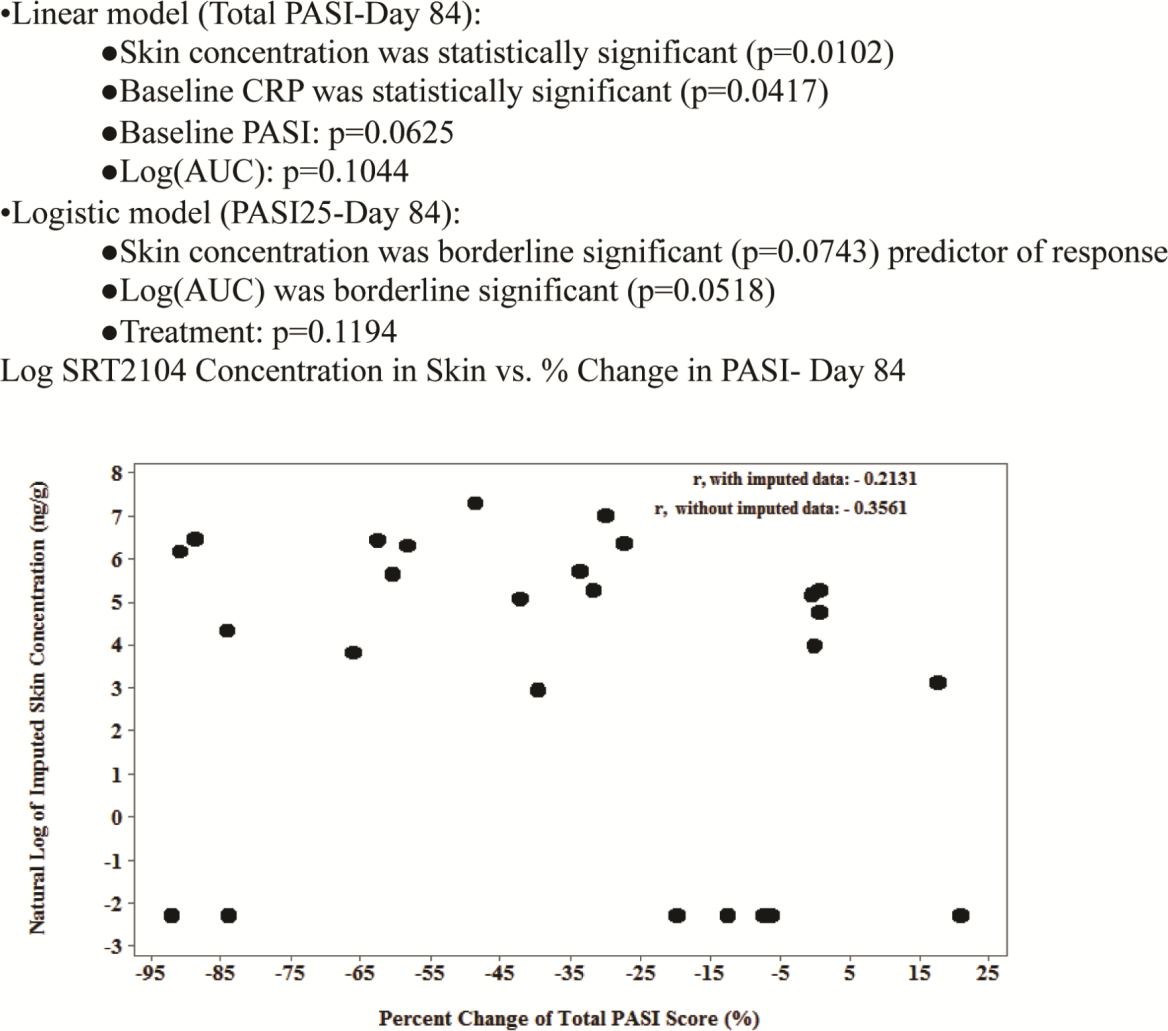
Post-hoc Exploratory Analyses of Predictors of Response.

Average exposure increased in a dose-dependent manner (S1 Table). Substantial inter-subject variability in SRT2104 exposure was observed (AUC %CV: 51–89%), as expected based on previous experience with the compound [11]. When categorized by the observed exposure, there was a marked difference in AUC and Cmax values in the low exposure vs. the high exposure group. Lesional biopsies assessed for SRT2104 levels demonstrated that SRT2104 was present in the skin (S1 Fig). Plasma levels of SRT2104 were proportional to SRT2104 concentrations in skin lesions (r = 0.48, p = 0.0118). While data on all subjects were not available and definitive conclusions could not be drawn, 100% of subjects that achieved a skin concentration ≥ 300 ng/g (natural log = 5.7) had improvement in their PASI scores (Fig 3). Post-hoc exploratory analyses suggest that skin concentration levels were predictive of improvement in PASI (Fig 3).

### Gene Expression Analysis in Skin Biopsies

**Fig 4:**
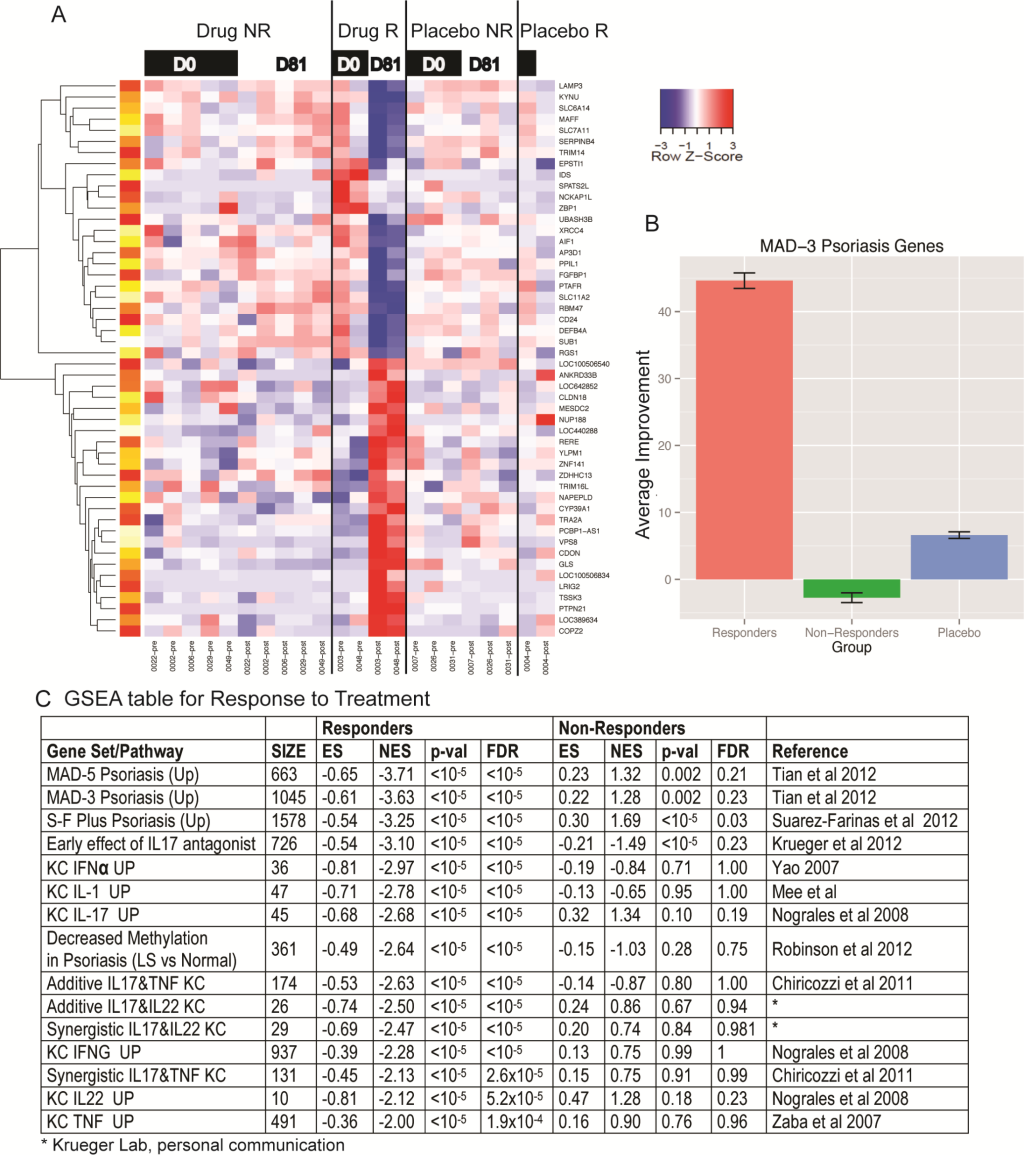
Microarray analysis in skin biopsies (A) Heat-map of genes modulated by drug treatment in psoriasis subjects (B) Average improvement after treatment for placebo, responders and non-responders (C) Gene set enrichment analysis for psoriasis transcriptome (Tian, Yao, Suarez-Farinas, Chircozzi, and Nograles) with respect to the IL-17/TNF-α synergistic phenotype, as defined by the synergistic increase.

We conducted global expression profiling in skin biopsies from placebo subjects, and drug responders (two subjects) vs. non responders (five subjects) at start of study day 1 and day 84 post treatment. The classification of drug responder vs. non-responder was based on histology as the endpoint. We identified a total of 123 probe sets (representing 77 unique known genes) that were modulated by drug treatment in responders (FDR<0.1, FC>2) (Fig 4), with 33 transcripts up-regulated and 90 transcripts down-regulated. In contrast, no differential expression induced by treatment was found in the drug-non responder and placebo groups at the chosen cut-off. Top differentially expressed genes in the responder group vs. non-responder and Placebo group in the study are presented in S3 Table. In responders, the changes in gene expression were highly related to dysregulated genes in the psoriasis population as quantified by gene set enrichment analysis (GSEA) (Fig 4C). Normalized enrichment score between -3.25 to -3.71 was observed for several transcriptomes of psoriasis vulgaris. Overall, there was an average 1.66-fold change towards recovery in psoriasis related gene transcripts (Fig 4), which represent a 45% improvement, with only slight improvement seen in the placebo-treated group. The GSEA approach also identified that gene changes were highly related to IL-17 regulated keratinocytes or to genes that are altered in psoriasis by an IL-17 antagonist. GSEA analysis showing impact on other pathways impacted by SRT2014 in the responder group are presented in S4 Table.

Amongst the genes impacted by drug treatment, Kynu, a gene implicated in tryptophan metabolism was significantly downregulated ~23 fold in the SRT2104 treatment group. Kynu is one of the most highly upregulated genes in psoriasis [13] and not modulated by etanercept treatment. As shown in Fig 3, other genes that were strongly suppressed by SRT2104 included the serine protease inhibitors (SERPINB4, SERPINB3, SERPINB13), members of the S100 family of proteins (S100A7, S100A12), SPRR2c, etc. previously shown to be highly upregulated in psoriasis [13]. SRT2104 caused ~15 fold reduction in the antimicrobial peptide S100A12 gene, a highly inflammatory molecule that binds to the receptor for advanced glycation end products and is increased in inflammatory dendritic cells and keratinocytes in response to inflammatory cytokines such as TNF-α, and IL-17 [5,14]. Further, drug treatment also reduced SPRR2c (~10 fold), another gene that plays a role in keratinocyte terminal differentiation [15,16]. Taken together, these results suggest that SRT2104 exerts its protective effects via modulation of multiple signaling pathways and by impacting keratinocyte differentiation.

### Safety

Thirty-nine subjects received treatment (7 placebo; 32 active). Three subjects in the placebo group (43%), 4 subjects (44%) in the 250 mg dose group, 9 subjects (75%) in the 500 mg group and 11 subjects (100%) in the 1000 mg group reported at least one AE (Table 4). The majority of AEs were mild to moderate in severity and self-limiting. The most frequently reported AEs in the SRT2104 arms were: dizziness, headache, psoriatic arthropathy, gastrointestinal symptoms and transaminase elevations. Serious adverse events (SAEs) were reported for 3 subjects (8%)– 2 subjects (22%) in 250 mg group (Pneumonitis, Pancreatitis) and 1 subject (9%) in 1000 mg group (ALT and Bilirubin increased). All SAEs were considered likely related or related to SRT2104. AEs leading to permanent discontinuation of active treatment occurred in 4 subjects (10%)–three of them being the ones described above having the SAEs; the fourth patient was in the 500 mg group and experienced increased hepatic enzymes.

**Table 4:** Summary of Adverse Events Occurring in >1 Subject^1^.

System Organ Class (any event), n (%)		SRT2104 Dose		
Preferred Term, n (%)^2^				
	Placebo (N = 7)	250 mg (N = 9)	500 mg (N = 12)	1000 mg (N = 11)
Subjects with Any Event (Total), n (%)	3 (43)	4 (44)	9 (75)	11 (100)
Dizziness	0	0	0	3 (27)
Headache	0	0	2 (17)	1 (9)
Psoriatic arthropathy	0	0	1 (8)	2 (18)
Upper respiratory tract infection	1 (14)	0	1 (8)	1 (9)
Alanine aminotransferase increased (ALT)	0	0	0	2 (18)
Aspartate aminotransferase increased (AST)	0	0	0	2 (18)
Fatigue	0	0	0	2 (18)
Hepatic enzyme increased	0	0	1 (8)	1 (9)
Nausea	0	1 (11)	0	1 (9)
Pain in extremity	0	0	1 (8)	1 (9)
Pruritus	1 (14)	0	0	1 (9)
Pyrexia	1 (14)	0	0	1 (9)
Vomiting	0	1 (11)	0	1 (9)

^1^ Data presented are number (%) of patients.

^2^ MedDRA 12.0.

## Discussion

SIRT1 activation represents a novel approach to managing inflammatory conditions via SIRT1’s action on precursors such as NF-κB and p53. In our trial, based on histopathology, we saw substantial improvement in 9 subjects following 84 days of treatment with SRT2104. Although absorption was relatively linear with dose, we did not observe a dose-response in the histology endpoint. A trend for dose/exposure was observed with the more classical psoriasis endpoints of PASI and PGA. The reasons for this are unclear but the observed high inter-patient variability in the exposure of SRT2104 and lack of detailed PK data across the course of the study may have been a factor.

While this study was not powered for formal hypothesis testing, the comparison of histological improvement against a historical placebo response rate of 5% was significant. An estimation approach was used for the direct comparison against the placebo arm in our trial because the study was not sufficiently powered to detect statistical differences; furthermore, as one patient responded to placebo within a small sample size, the historical rate was used to prevent an overestimation of the placebo effect. If we assume 1 in 10 patients respond to placebo, we will need 15(19) patients per group to detect significance differences between rate of response of SRT2104 250mg and Placebo using a 1-tail (2-tail) fisher’s exact test; likewise, for the rate of the 500 mg group, 19(24) patients will be needed. Concern about the small sample size led to the decision to prospectively define the historical placebo response rate of 5% as the primary comparator for analysis for this trial. Given the small sample size, it is unclear whether the proportion of placebo responders seen in our small trial would be replicated in a larger experiment. PASI, PGA and gene expression data all supported the primary histological endpoint.

The role of TNFα and IL-17 in psoriasis pathogenesis is well documented [5,17,18]. In the current study, transcriptomic analysis revealed that SRT2104 significantly reduced known IL-17 and TNFα responsive genes including SERPINB4, S100A12, SERPINB3, kynu etc. even though the sample size for this analysis was small. Keratinocytes are a key driver of pathogeneic inflammation through integrating responses to TNFα and IL-17. SRT2104 treatment suppressed keratinocyte differentiation relevant genes including SPRR2c, SERPINB4, and SERPINB3. One of the most highly modulated genes by SRT2104 included Kynu that regulates tryptophan metabolism. Although the precise role of this pathway in psoriasis is unknown, upregulation of tryptophan catabolism has been shown to confer antibacterial effector functions [17]. Interestingly kynu is part of the etanercept molecular scar signature that is not modulated by etanercept therapy even though clinical efficacy is achieved. Possibly, SRT2104 may be modulating the lipid barrier of the epidermis of psoriatic skin via modulation of keratinocyte differentiation genes, which would be consistent with the observed improvement in skin histology. These results indicate a combinatorial effect of SRT2104 on TNFα, and IL-17 inflammatory signaling pathways and keratinocyte differentiation that could be a contributing factor towards improvement in clinical scores by the SIRT1 activator, SRT2104.

Exposure for 84 days is the longest treatment duration with SRT2104 to-date. All dose levels were well tolerated and no dose-limiting toxicities were observed which would have curtailed dose escalation; however, only subjects treated with SRT2104 withdrew from treatment prior to completing the 84 day regimen. Adverse events were infrequent and typically mild to moderate and included gastrointestinal symptoms, headaches, dizziness, psoriatic arthropathy and transaminase elevations.

Pharmacokinetics were modeled based on a sparse sampling approach. As has been observed in other studies with SRT2104 [11], inter-subject variability was high (AUC %CV > = 50%:) leading us to examine clinical activity using exposure groupings as well as dose level, although in this study average exposure increased in a dose-dependent, but not fully proportional, manner. Because higher levels of SRT2104 in psoriatic lesions were predictive of improvement in PASI, more predictable absorption, and thus more predictable SRT2104 concentration in the skin, could lead to a more predictable level of response.

The armamentarium of approved treatments for moderate-to-severe psoriasis includes agents with serious potential side-effects and substantial inconvenience, suggesting that a well tolerated orally-administered treatment could have a place in the therapeutic options for subjects with plaque-type psoriasis. Given the interesting signals of clinical activity, impact on gene expression and the generally favorable safety profile seen in this study, further investigation of SIRT1 activators for the treatment of psoriasis is warranted.

## Supporting Information

S1 CONSORT Checklist CONSORT Checklist. (DOC)

S1 Fig SRT2104 Plasma: Skin Concentration. (TIF)

S1 Protocol Clinical study protocol. (PDF)

S1 Table SRT2104 Pharmacokinetic Parameters by Treatment and Exposure groups. (DOCX)

S2 Table Proportion of Subjects Improvement Based on PGA Score–Day 84. (DOCX)

S3 Table Top Differentially Expressed Genes. (DOCX)

S4 Table GSEA Analysis of the Responder Group. (DOCX)
