# Supplementary figures and images for: A Randomized, Placebo-Controlled Study of SRT2104, a SIRT1 Activator, in Patients with Moderate to Severe Psoriasis
Source: PLoS One. 2015 Nov 10;10(11):e0142081. doi: 10.1371/journal.pone.0142081 (PMC4640558; doi:10.1371/journal.pone.0142081)

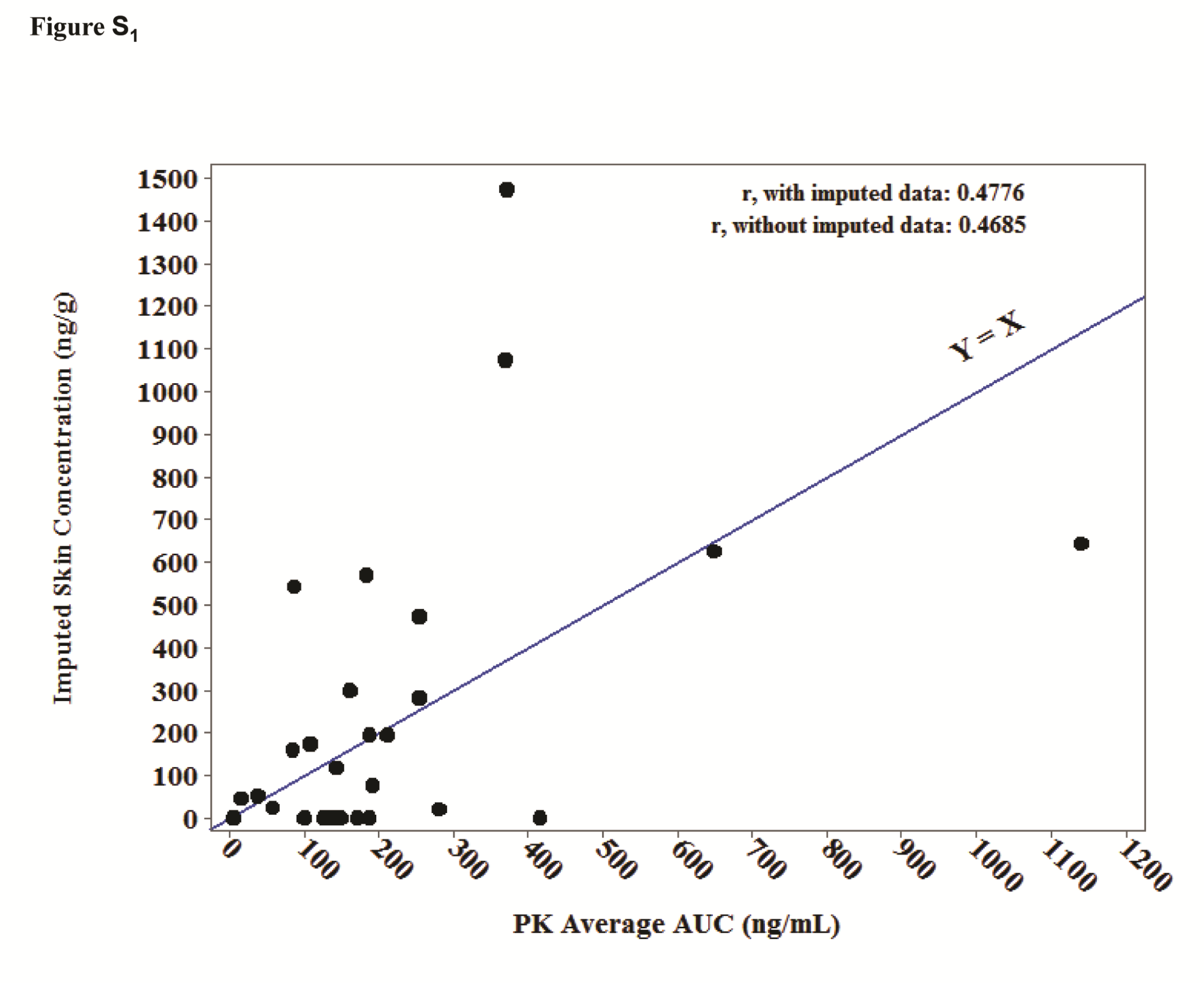

Supplement: S1 Fig — (TIF) [file pone.0142081.s002.tif]
