# Supplementary material for: A Randomized, Placebo-Controlled Study of SRT2104, a SIRT1 Activator, in Patients with Moderate to Severe Psoriasis
Source: PLoS One. 2015 Nov 10;10(11):e0142081. doi: 10.1371/journal.pone.0142081 (PMC4640558; doi:10.1371/journal.pone.0142081)
Supplement: S1 Protocol — (PDF) [file pone.0142081.s003.pdf]

CLINICAL STUDY PROTOCOL

A Randomized, Double-Blind, Placebo-Controlled, Phase IIa Study of the Clinical Activity, Safety, and Tolerability of SRT2104 in Subjects with Moderate to Severe Plaque-Type Psoriasis

Protocol Number: SRT-2104-013

Indication: Plaque-Type Psoriasis

Phase: IIa

Design: Randomized, Double-Blind, Placebo-Controlled, Dose-Escalation

Sponsor: Sirtris Pharmaceuticals, Inc.

IND Number: 108034

Investigational Product: SRT2104

Version: 3.0

Protocol History

Original, Version 1.0	February 12, 2009
Original, Version 2.0	March 19, 2010
Amendment 1.0	October 22, 2010

Sirtris Pharmaceuticals, Inc.
200 Technology Square
Cambridge, MA 02139 USA
Telephone: +1 (617) 252-6920

Approved by:

Eric W Jacobson MD (signature) | 22 OCT 2010

Eric W. Jacobson, MD, Chief Medical Officer | Date

Confidentiality Statement
The information contained herein is confidential and the proprietary property of Sirtris Pharmaceuticals, Inc. and any unauthorized use or disclosure of such information without the prior written authorization of Sirtris Pharmaceuticals, Inc. is expressly prohibited.

1 PROTOCOL SUMMARY

Study Title: A Randomized, Double-Blind, Placebo-Controlled, Phase IIa Study of the Clinical Activity, Safety, and Tolerability of SRT2104 in Subjects with Moderate to Severe Plaque-Type Psoriasis

Number of Study Center(s): Approximately five centers located in the United States are planned for this study.

Study Phase: Phase IIa

Study Period: Approximately 18 weeks for each dose cohort

Study Objectives:

Primary:

1. To assess the effects of 250 mg, 500 mg, and 1000 mg SRT2104 on clinical activity in subjects with moderate to severe plaque-type psoriasis based on histological assessment of skin biopsies after 12 weeks of exposure
2. To assess the safety and tolerability of multiple doses of SRT2104 in subjects with moderate to severe plaque-type psoriasis

Secondary:

1. To assess the effects of SRT2104 on the Psoriasis Area and Severity Index (PASI) in subjects with moderate to severe plaque-type psoriasis after 4, 8, and 12 weeks of exposure
2. To assess the effects of SRT2104 on the Physician's Global Assessment (PGA) score in subjects with moderate to severe plaque-type psoriasis after 4, 8, and 12 weeks of exposure
3. To determine the pharmacokinetics of 84 days of dosing with 250 mg, 500 mg and 1000 mg SRT2104 in the fed state in subjects with moderate to severe plaque-type psoriasis
4. To assess the pharmacodynamic effects of SRT2104 in subjects with moderate to severe plaque-type psoriasis

Exploratory:

1. To characterize expression patterns of select genes and proteins hypothesized to be involved in psoriasis pathophysiology and sirtuin pathways and to evaluate the relationship between these biomarkers and investigational product pharmacokinetics and/or clinical activity
2. To assess the effects of SRT2104 on sense of depression and anxiety in subjects with moderate to severe plaque-type psoriasis after 4, 8, and 12 weeks of exposure using the Patient Health Questionnaire-9 (PHQ-9) and the Hospital Anxiety and Depression Scale (HADS)
3. To assess the effects of SRT2104 on health-related quality of life in subjects with moderate to severe plaque-type psoriasis after 12 weeks of exposure using the Koo-Menter Psoriasis Instrument, PQOL-12.

Study Design:
This phase IIa, proof of concept, randomized, double-blind, placebo-controlled, dose-escalation study will be conducted in approximately 30 subjects with moderate to severe plaque-type psoriasis. For each dose cohort there will be a screening period, a 12-week treatment period with 7 on-treatment visits (Days 1, 14, 28, 42, 56, 70 and 84) and a follow-up safety assessment (Day 114).

Three cohorts of ten subjects each will be enrolled. Subjects within each cohort will be randomized 4:1 to receive SRT2104 at one of three escalating doses (250, 500, or 1000 mg/day) or placebo. Each cohort of subjects will be dosed sequentially. Dosing in the second and third cohort will not commence until subjects in the previous cohort have completed at least 28 days of dosing, and a review of key safety parameters has been completed by an Internal Safety Review Committee (ISRC).

Subjects who provide informed consent will undergo screening procedures within 21days of randomization. Subjects will be enrolled and randomized into the study on approximately Day – 6 and receive investigational product on Day 1. Subjects will take blinded investigational product on a daily basis from Day 1 through Day 84.

For the safety evaluation, adverse events (AEs) will be monitored from Day 1 through a follow-up visit that will occur 30 days after discontinuation of investigational product on Day 84. Vital signs, clinical laboratory results (hematology, chemistry, urinalysis), ECGs and physical examinations will be assessed at periodic intervals from Day 1 through Day 84.

Skin biopsies of the same designated psoriatic lesion will be conducted on Days 1 and 84 to assess the effects of SRT2104 on histologic markers of inflammation. Disease assessments using the PASI score and the PGA will be conducted on Days 1, 28, 56, 84 and 114 to quantify the effects of SRT2104 on psoriasis activity.

Sense of well-being will be assessed using depression and anxiety scales (PHQ-9 and HADS respectively) which will be completed on Days 1, 28, 56 and 84.

Quality of Life (QOL) will be assessed on Days 1 and 84 using the PQOL-12.

Blood will be obtained on Days 28, 56 and 84 for pharmacokinetic (PK) and pharmacodynamic (PD) assessments.

Subjects who prematurely discontinue from the study will complete Day 84 assessments at the time of discontinuation.

Number of Subjects: Approximately 30 subjects will be enrolled.

**Duration of Subjects Participation/Duration of Study/Duration of Treatment:**<br>Subject participation will include a screening period of up to 21 days and an 84-day dosing period. Subjects will return to the clinic 30 days after their last dose of investigational product for a follow up visit. The duration of each subject's participation is expected to be approximately 18 weeks. The study duration (first subject's first visit through last subject's last visit) is anticipated to last approximately 10 - 12 months.
**Drug Supply, Dosage, and Mode of Administration:**<br>Subjects will be randomized 4:1 to receive active SRT2104 at one of three dose levels (250, 500, or 1000 mg/day) or placebo. Investigational product will be supplied as 250 mg capsules of SRT2104 along with matching placebo capsules that will be administered orally once daily for 84 consecutive days. The subjects and the investigator will be blinded to active vs. placebo treatment assignment. Dosing with SRT2104 or placebo is to take place at approximately the same time every day, approximately 15 minutes following consumption of food. Subjects must wait at least 1 hour after dosing before consuming additional calories.

CRITERIA FOR EVALUATION

Safety and Tolerability:
The incidence of AEs and clinically significant abnormal laboratory values will be recorded based upon investigator observation and subject reporting. Safety will be monitored by reports of AEs (at all visits after the first dose has been administered through 30 days after the last dose), vital sign measurements, physical examinations, laboratory parameters and electrocardiograms. Concomitant medications and AEs will be recorded at every visit. Additional visits will be permitted for safety follow-up as required.

Pharmacokinetics:
Blood will be obtained using a sparse sampling technique for PK and PD assessments.

Pharmacodynamics:
Biomarkers for psoriatic disease activity and/or sirtuin pathway activation will be analyzed in blood samples collected for this purpose and may include, but may not be limited to, hsCRP and FGF21.

Activity:
The primary clinical activity endpoint is change in histologic assessments of skin biopsies of psoriatic lesions from baseline to 12 weeks. Skin biopsy samples will be evaluated for general appearance, epidermal thickness, total inflammatory infiltrate, specific cell numbers (including but not limited to CD163+ monocytes, CD11c+ dendritic cells, CD83+and/or CD206+ cells, and CD3+ T-cells), and keratinocyte expression of K-16 and ICAM-l.

Secondary clinical activity endpoints are PASI-50 and PASI-75 response rates, defined as the proportion of subjects who achieve a PASI-50 or PASI-75, mean change in PASI score, proportion of subjects who achieve “clear” or ”almost clear” on the PGA assessment, and proportion of subjects who achieve improvement in PGA by one or more levels.

RT-PCR will be used to assess expression of specific genes at baseline and after 12 weeks which may include, but not be limited to, K-16, IL-1β, IL-6, IL-8, IL-10, IL-12, IL-17, IL-22, IL-23, INFγ, TNF-α, iNOS, IL-1R antagonist, PGC-1α, NCoR, NFκβ, FOXO, p300, PPAR alpha, PPAR delta, and p53. In addition, global changes in gene expression may be assessed using gene micro-array techniques.

Exploratory clinical activity endpoints include assessments of the subject’s sense of well-being (PHQ-9 and HADS) and health-related quality of life (PQOL-12).

General Analysis Plan:

The primary objectives are (1) to assess the effects of SRT2104 on clinical activity, based on the Krueger criteria, relative to a historical placebo response rate of 5% and (2) to assess safety and tolerability of SRT 2104. The first objective will be accomplished based on an exposure-response analysis. Previous studies have indicated that the pharmcokinetic exposure is relatively variable. For this reason, the active treatments will be combined and then dichotomized into low and high drug exposure groups. The cut-off point will likely be the midpoint of exposure but the selection of the final cut-off point will be dependent on the distribution of exposure. Point estimates and 90% confidence intervals will be constructed for the differences between the proportion of responders, defined as “good” or “excellent” Krueger improvement score, for each of the exposure groups and the historical null placebo response of 5%. No formal hypothesis testing will be performed. The second primary objective will be accomplished by review of individual subject data and descriptive summaries of the safety data. Safety displays will be summarized by treatment group (placebo, 250mg, 500mg, and 1000mg)

Point estimates and 90% confidence intervals will also be constructed for the secondary endpoints of clinical activity (PASI-50, PASI-75, and PGA of “almost clear” or “clear”) for the differences between the proportion of responders for each of the exposure groups and the historical null placebo response of 5%. The clinical activity endpoints will also be summarized by treatment group, as a secondary analysis. All other pharmacodynamic endpoints will summarized by treatment group. Point estimates and corresponding 90% confidence intervals will be constructed for PK comparisons. A repeated measures analysis of covariance using baseline as a covariate will be performed on the PASI response.

Safety evaluations will be based on the incidence, severity, and type of AEs and clinically significant changes in the subject’s physical examination findings, vital signs, and clinical laboratory results. Safety variables will be tabulated and presented for all subjects who receive SRT2104 or placebo (the safety population). Exposure to investigational product and reasons for discontinuation of study treatment will be tabulated.

Listing of individual subject plasma SRT2104 concentrations and blood sampling times will be prepared. Pharmacokinetic data will be presented in graphical and/or tabular form and will be summarized descriptively.

Rationale for Number of Subjects:

Sample size is based on feasibility. A sample size of 8 evaluable subjects per active treatment group and 6 evaluable placebo subjects in a merged placebo group will be assessed. Subjects who withdraw from the study prior to 8 weeks of treatment may or may not be replaced (see Section 5.2.2).

1.1 Schedule of Events

A full description of the study procedures and applicable visit windows is provided in Section 6.

Study Period	Screening	Dosing Period							Follow Up
Visit Number	1	2	3	4	5	6	7	8	
Study Day(s)	-21 to -6	1[8]	14	28	42	56	70	84[10]	114
Clinic Visit	X	X	X	X	X	X	X	X	X
Informed Consent	X								
Medical History[1]	X								
Viral Serology	X								
Photographs (total body surface area involvement)		X		X		X		X	
Randomization[2]	X								
Investigational Product Dispensed		X		X		X			
Physical Examination[3] (including height at Screening and weight at all subsequent visits)	X	X		X		X		X	X
Vital Signs	X	X	X	X	X	X	X	X	X
Chest X-Ray	X								
12-lead ECG	X	X		X		X		X	
Clinical Chemistry/Hematology[4]	X	X	X	X	X	X	X	X	X
Urinalysis	X	X	X	X	X	X	X	X	X
Pregnancy Test[5]	X	X		X		X		X	X
Tuberculosis Test	X								
Adverse Event/Concomitant Medication Assessment		X	X	X	X	X	X	X	X
PK Sampling[6]				X		X		X	
PASI & PGA		X		X		X		X	X
QOL assessments (PHQ-9, HADS, PQOL-12[7])		X		X		X		X	
Biomarkers		X		X		X		X	
Pharmacogenetics Sample[9]		X							
Skin Biopsy (Immunohistochemistry and Gene Expression Profiling)		X						X	

Footnotes:

1. Medical events occurring prior to the first dose will be collected on the medical history case report form (CRF). AEs occurring after the first dose will be recorded on the AE CRF. AEs and concomitant medications will be followed for 30 days after the last dose of study medication.
2. Subjects will be randomized to a treatment on approximately Day – 6 to allow sufficient time for delivery of investigational product.
3. A complete physical examination (PE) will be conducted at the Screening Visit. A symptom-driven, directed PE will be performed as needed at all other timepoints.
4. See Section 6.3 for a complete listing of safety lab parameters to be measured/analyzed. Lipid profiling and coagulation studies will be performed in fasting samples at Screening, and on Days 1, 42 and 84 only.
5. Serum pregnancy test to be performed at Screening; urine screen for pregnancy at all other timepoints.
6. PK sampling will be performed according to the schedule described in Section 6.2.
7. PQOL-12 to be performed on Days 1 and 84 only
8. The investigator may at his/her discretion conduct a portion of the assessments scheduled for Day 1 on Day -1.
9. See Appendix 2 for a description of the pharmacogenetic assessment.
10. Subjects withdrawing from the study prior to the study assessments on Day 84 will undergo all Day 84 assessments and return for a follow-up visit 30 days following the last dose of investigational product.

1.2 Schematic Diagram of Study Design

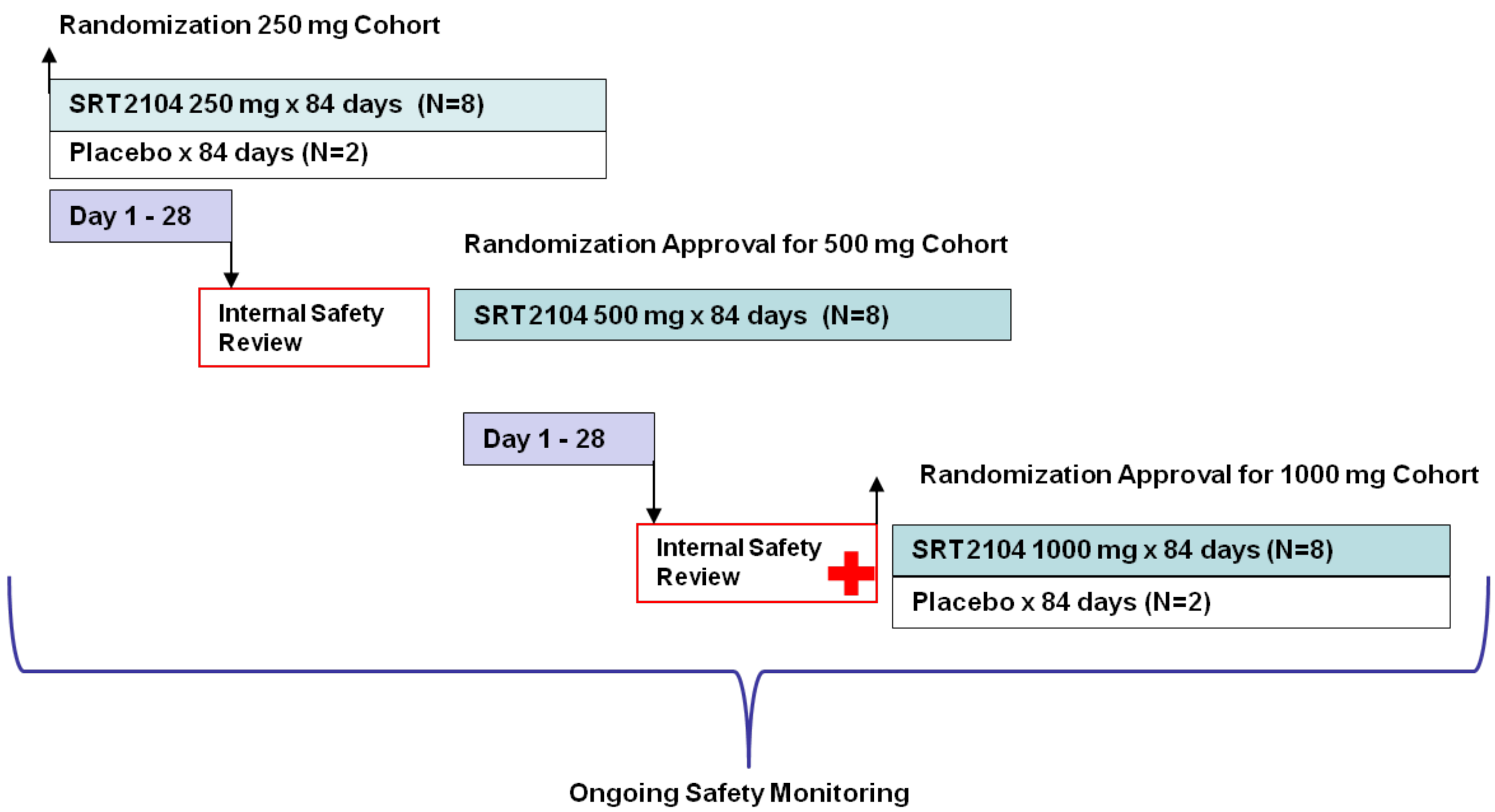

TABLE OF CONTENTS

1 PROTOCOL SUMMARY 2
1.1 Schedule of Events 7
1.2 Schematic Diagram of Study Design 8
LIST OF ABBREVIATIONS AND GLOSSARY OF TERMS 12
2 INTRODUCTION AND STUDY RATIONALE 15
2.1 Disease Background 15
2.2 Scientific Background 15
2.3 SRT2104 Non-Clinical Experience 16
2.4 SRT2104 Clinical Experience 18
3 STUDY RATIONALE 22
4 STUDY OBJECTIVES 23
4.1 Primary 23
4.2 Secondary 23
4.3 Exploratory 23
5 INVESTIGATIONAL PLAN 24
5.1 Overall Study Design 24
5.2 Selection of Study Population 24
5.2.1 Number of Subjects 24
5.2.2 Replacement of Subjects 24
5.2.3 Inclusion Criteria 24
5.2.4 Exclusion Criteria 25
5.2.5 Definition of Treatment Failure 26
5.2.6 Removal of Subjects from the Study 26
5.3 Selection of Doses in the Study 27
5.4 Identity of Investigational Product 28
5.5 Treatments to be Administered 28
5.6 Method of Assigning Subjects to Treatment Groups 29
5.7 Individuals Who Will Be Unblinded to Treatment Assignments 29
5.8 Unblinding Procedures 29
5.9 Duration of Treatment 30
5.10 Internal Safety Review Committee 30
5.11 Safety Review and Dose Escalation or Cessation 30
5.11.1 Criteria for discontinuing dosing/dose escalation 30
5.12 Prior Treatments 31
5.13 Proscribed Medications 31
5.14 Permitted Medications 31
5.15 Contraceptive Use 32
5.16 Treatment Compliance 33
5.17 Missed Doses of Investigational Product 33
5.18 Schedule of Events 33
6 STUDY PROCEDURES 34
6.1 Study Procedures 37

6.1.1 Visit 1 (Screening) 37
6.1.2 Visit 2 37
6.1.3 Visits 3, 5 and 7 38
6.1.4 Visits 4 and 6 38
6.1.5 Visit 8 38
6.1.6 Follow-Up Procedures 38
6.2 Pharmacokinetic Sampling 39
6.3 Laboratory Assessments 39
6.4 Skin Biopsy Evaluation and Scoring 41
6.5 Appropriateness of Measurements 41
6.6 Data Quality Assurance 42

7 ADVERSE EVENTS 43
7.1 Definitions 43
7.1.1 Adverse Event Definition 43
7.1.2 Serious Adverse Event Definition 43
7.1.3 Suspected Unexpected Serious Adverse Reaction Definition 45
7.1.4 QTc Withdrawal Criteria 45
7.1.5 Liver Chemistry Stopping Rules and Follow-up 45
7.2 Procedures for Recording and Reporting AEs and SAEs 49
7.3 Monitoring of Adverse Events and Period of Observation 52
7.4 Pregnancy Reporting 53

8 STATISTICAL PROCEDURES 54
8.1 Randomization and Stratification 54
8.2 Sample Size 54
8.3 Populations for Analysis 55
8.4 Procedures for Handling Missing, Unused, and Spurious Data 56
8.5 Statistical Methods 56
8.5.1 Subject Disposition 56
8.5.2 Subjects Baseline Characteristics 57
8.5.3 Exposure to Investigational Product 57
8.5.4 Safety Analysis 57
8.5.5 Pharmacokinetic and Pharmacokinetic/Pharmacodynamic Analyses 58
8.5.6 Activity Analysis 59
8.6 Procedures for Reporting Deviations to Original Statistical Analysis Plan 60

9 ADMINISTRATIVE REQUIREMENTS 61
9.1 Good Clinical Practice 61
9.2 Ethical Considerations 61
9.3 Subject Information and Informed Consent 61
9.4 Subject Confidentiality 61
9.5 Protocol Compliance 62
9.6 Study Monitoring 62
9.7 On-site Audits 62
9.8 Case Report Form Completion 63
9.9 Drug Accountability 63
9.10 Premature Closure of the Study 64

9.11 Record Retention 64
9.12 Sample Disposition 64
9.13 Liability and Insurance 65

10 USE OF INFORMATION 66

11 INVESTIGATOR AGREEMENT 67

12 REFERENCES 68

13 Appendix 1 List of Permitted Rescue Medications 71

14 Appendix 2 Pharmacogenetic Research 72

15 Appendix 3 Summary of Changes to Amendment 1 75

LIST OF ABBREVIATIONS AND GLOSSARY OF TERMS

AE	Adverse Event
ALT	Alanine Aminotransferase
ANCOVA	Analyses of Covariance
AUC	Area Under the Plasma Concentration Time Curve
°C	Degrees Celsius
CFR	Code of Federal Regulations
CL	Clearance
C_{max}	Maximum (plasma) Concentration
CR	Calorie Restriction
CRP	C-reactive Protein
CRF	Case Report Form
CRO	Contract Research Organization
CTC	Common Toxicity Criteria
DIO	Diet-Induced Obesity
DNA	Deoxyribonucleic Acid
DSS	Dextran Sulphate Sodium
EAE	Experimental Autoimmune Encephalitis
ECG	Electrocardiogram
FAS	Full Analysis Set
FDA	Food and Drug Administration (U.S.)
FGF21	Fibroblast Growth Factor 21
FOXO	Forkhead Transcription Factors
GCP	Good Clinical Practice
GSK	GlaxoSmithKline
HADS	Hospital Anxiety and Depression Scale
HCV	Hepatitis C Virus
HIV	Human Immunodeficiency Virus
hr	Hour
hsCRP	High sensitivity C-reactive protein
ICH	International Conference on Harmonization

IEC	Independent Ethics Committee
IL	Interleukin
IRB	Institutional Review Board
ISRC	Internal Safety Review Committee
IV	Intravenous
Km	Michaelis-Menten Constant
LOCF	Last Observation Carried Forward
LPS	Lipopolysaccharide
MedDRA	Medical Dictionary for Regulatory Activities
NCI	National Cancer Institute
NCoR	Nuclear Receptor Co-repressor
NFκβ	Nuclear Factor Kappa-Light-Chain-Enhancer of Activated B Cells
NOAEL	No Observable Adverse Effect Level
ob/ob	Genetically Obese Mouse Model, $Lep^{ob/ob}$
PASI	Psoriasis Area and Severity Index
PD	Pharmacodynamics
PGA	Physician’s Global Assessment
PGC-1α	PPAR Gamma Coactivator-1α
PGx	Pharmacogenetics
PHQ-9	Patient Health Questionnaire
PINP	Procollagen-I-N-Terminal Propeptide
PK	Pharmacokinetics
PPS	Per-Protocol Analysis Set
PQOL-12	Koo-Menter Psoriasis Quality of Life Instrument
PUVA	Psoralen and Ultraviolet Light A
QOL	Quality of Life
SAE	Serious Adverse Event
SAF	Safety Analysis Set
SIRT	Silent Information Regulator Transcript; Sirtuin
SIRT1	Sirtuin Enzyme 1
SRT2104	A SIRT1 Activator

SD	Standard Deviation
SUSAR	Suspected Unexpected Serious Adverse Reaction
t	Time
Tmax	Time of Maximum Concentration
$t_{1/2}$	Terminal Elimination Half –Life
TGF	Transforming Growth Factor
TMF	Trial Master File
TNF	Tumor Necrosing Factor
ULN	Upper Limit of Normal
UVB	Ultraviolet light B
V	Volume of distribution
WBC	White Blood Cell

2 INTRODUCTION AND STUDY RATIONALE

2.1 Disease Background

Psoriasis is a chronic inflammatory skin disorder that impacts approximately 2-3% of the world's population. [Schon, 2005; Koo J, 1996]. It is typically characterized by sharply demarcated, raised, erythematous plaques covered by silvery white scales. Psoriasis impacts both the physical and emotional well-being of the patient. Its effect on quality of life is comparable to that of other chronic medical disorders [Rapp, 1999]. Significant unmet medical need remains for safe, effective, and convenient treatments.

Psoriasis is a complex, immune-mediated disease in which T-lymphocytes, neutrophils, and dendritic cells play a major role. Numerous cytokines are over-expressed in psoriatic skin lesions including TNF-α, IL-17, and IL-23 [Krueger JG, 2007]. Several growth factors are also over-expressed including transforming growth factor type alpha (TGF-α). The result is an epidermis characterized by hyperproliferation, inflammatory cell infiltrates, and vascular changes. Current treatments are primarily geared at reducing the excessive cellular proliferation and/or blocking the inflammatory response.

2.2 Scientific Background

One novel therapeutic approach to treating psoriasis and other diseases of inflammation has come from the study of aging and calorie restriction (CR). CR is a dietary regimen in which 30%-40% fewer calories than those required to maintain ideal body weight are consumed. It has been demonstrated that CR extends lifespan in lower organisms and mammals and improves a number of metabolic and inflammatory parameters [Heilbronn, 2003; Roth, 2001, Fontana, 2009]. As such the molecular components of the aging pathways downstream of CR may provide relevant intervention points for the development of therapeutic drugs to treat inflammatory and metabolic disease [Weindruch, 2001; Ingram, 2006].

Sirtuin Enzyme 1 (SIRT1) is a member of the sirtuin family of NAD^+-dependent deacetylases [Frye, 1999; Frye, 2000; Imai, 2000]. There are seven human sirtuins (SIRT1-7) with different subcellular compartmentalization and downstream targets [Blander, 2004]. SIRT1 is predominantly nuclear and is upregulated in tissues of calorically restricted animals [Cohen, 2004]. The precise biological pathway whereby SIRT1 promotes the beneficial effects of CR is an area of intense study, but it appears that the ability of SIRT1 to interact and deacetylate PGC-1α is an important component [Rodgers, 2005]. PGC-1α controls energy metabolism and muscle function with an inhibitory role in pro-inflammatory cytokine production [Handschin, 2008] and has been implicated as a key mediator of the effects of CR [Corton,

2005]. Additionally, a number of other cellular substrates for SIRT1 have been identified including NCoR, p300, NFκβ, FOXO, and p53 [Bouras, 2005; Brunet, 2004; Luo, 2001; Motta, 2004; Nemoto, 2005; Picard, 2004; Rodgers, 2005; van der Horst, 2004; Vaziri, 2001; Yeung, 2004]. For example, SIRT1 has been shown to physically interact with and deacetylate the RelA/p65 subunit of NFκβ, and thereby inhibit NFκβ-induced transcription [Yeung, 2004; Sirtris unpublished data] which is involved in up regulation of proinflammatory cytokines such as TNF-α and IL-6. Furthermore, SIRT1 activators have been shown to inhibit TNF-α secretion in both in vitro [Smith 2009; Yoshizaki 2009] and in vivo (Sirtris unpublished data) LPS-induced cellular and animal models. Through modulation of the activities of these proteins, SIRT1 regulates inflammation, cellular differentiation and survival, mitochondrial biogenesis, and glucose metabolism [Cohen, 2004; Heilbronn, 2005; Nisoli, 2005; Picard, 2004].

SRT2104 is a potent and selective small molecule activator of SIRT1. SRT2104 was identified as a SIRT1 activator in a high throughput screen of a diverse library of 290,000 compounds [Milne, 2007]. SIRT1 activity is increased by SRT2104 due to a lowering of the K_m for its acetylated protein substrate, resulting in an approximately two-fold increase in activity. SRT2104 is selective for SIRT1 activation over the two most closely related sirtuin homologs, SIRT2 and SIRT3.

2.3 SRT2104 Non-Clinical Experience

The effect of once daily oral administration of SRT2104 on fasting blood glucose and fed insulin levels, body weight, triglyceride and plasma lipid levels was evaluated in a number of animal models of diabetes and obesity (DIO mice and *ob/ob* mice). In general, SRT2104 lowered fasting blood glucose and fed insulin and enhanced the response to a glucose tolerance test. No effect on body weight was observed with SRT2104. Activity in the diabetes models was seen following daily doses of 100-3000 mg/kg/day of SRT2104. SRT2104 has also shown efficacy following once daily oral administration in a number of *in vivo* inflammation models including LPS-induced TNF-α production, dextran sulphate sodium (DSS) - and trinitrobenzesulphonic acid-induced colitis, cecal ligation and puncture-induced sepsis as well as in experimental autoimmune encephalomyelitis (EAE). The efficacy of SRT2104 in these inflammation studies was seen at doses of 10 – 300 mg/kg/day given orally once daily for five to twenty eight days depending on the model studied. In the DSS and EAE models SRT2104 was given therapeutically, that is after the diseases had been induced in the mice.

SRT2104 was tested *in vitro* in counter-receptor binding studies and showed no significant inhibitory activity against 39 common receptors. SRT2104 was not an inhibitor of five major cytochrome P450

isoforms tested (CYP1A, CYP2C9, CYP2C19, CYP2D6 and CYP3A4), and is not a significant inducer of cytochrome P450 isoforms CYP1A and CYP3A4 at the concentrations tested.

The non-clinical safety of SRT2104 was investigated in the Ames and mouse micronucleus genetic toxicology models, and in safety pharmacology studies in rats and dogs. SRT2104 was not genotoxic at the doses investigated. No central nervous system (CNS), cardiovascular system (CVS), or pulmonary effects were observed in the safety pharmacology studies at the doses tested (300-2000 mg/kg).

SRT2104 has been dosed up to 2000 mg/kg/day in two species (rat and dog) for 28 days. The compound was well tolerated at all doses in both species. Toxicity studies showed a no observable adverse effect level (NOAEL) of 2000 mg/kg/day in male rats and 1000 mg/kg/day in female rats and 1000 mg/kg/day in male and female dogs. In the male rat, the NOAEL was 2000 mg/kg/day, the highest dose tested. In the female rat, the NOAEL was considered to be 1000 mg/kg/day, due to a vacuolation of pancreatic acinar cells in 3 of 10 animals on Day 29 which was not seen after 4 weeks in the recovery animals. The physiological significance of this finding is unclear. Furthermore, this effect was not seen in the rat 90 day study or in the 28 or 90 day dog studies. The NOAEL in the female dog was determined to be 1000 mg/kg/day due to a mild increase in indirect bilirubin in the 2000 mg/kg/day group and microscopic bilirubin accumulation in the liver of one female dog at 2000 mg/kg/day. No adverse findings were seen in male dogs.

SRT2104 has also been dosed in two species (rat and dog) for 90 days. In the 90 day studies no toxicologically relevant adverse events were seen at the highest doses tested in rats (2,000 mg/kg/day males, 1,000 mg/kg/day females) and in dogs (300 mg/kg/day in fed males and females). In the 90 day rat study there were reductions in mean body weights primarily due to a reduction in food consumption during the first week of the study. There were reversible increases in bilirubin in both male and female rats with no corresponding histopathological changes in the liver. Minor histopathology findings of increased acinar cell apoptosis in the pancreas and accumulation of amorphous material in the basal pits of the stomach were judged to be non-adverse since neither were associated with overt degenerative changes and/or were also seen in control rats.

In the 90 day dog study there were mild, reversible increases in serum total/direct/indirect bilirubin in males and females at 300 mg/kg/day and reversible mild increases in serum alkaline phosphatase (males) or cholesterol (females) at 300 mg/kg/day. The increased serum bilirubin levels corresponded with pigment accumulation in the canaliculi of the liver on Day 91 for the 100 and 300 mg/kg/day males and

females. However, the pigment accumulation and increased serum bilirubin, suggestive of impaired excretion or stasis, were not associated with any microscopic effect (degeneration or necrosis) on bile duct epithelium or hepatocytes. Furthermore, the clinical pathology results were reversible and well within the normalized historical control ranges. The findings seen in the 90 day studies reflect what was seen in the 28 day studies with an increase in bilirubin being the most notable finding in both the dog and rat studies. The stasis finding at 2000 mg/kg/day in female dogs was judged to be adverse in the 28 day study but not adverse in the 90 day study due to the observation that there was no progression from 28 to 90 days and no evidence of necrosis, inflammation or any damage associated with the stasis.

From a safety perspective, the 1000 mg/day dose, the highest dose to be tested in this SRT-2104-013 protocol, is supported by the pre-clinical safety toxicology package and by the cumulative human safety experience gathered to date. From the pre-clinical 90-day toxicology studies, the safety intervals are 1.3-3.6 based on C_{max} and 1.9-6.8 based on AUC at the NOAELs. There were no adverse findings at the highest doses tested in the 90-day studies so these safety intervals may be an under-estimate of the true values.

2.4 SRT2104 Clinical Experience

As of December 31 2009, approximately 85 subjects have been dosed in completed clinical trials with SRT2104. Based on currently available clinical data, the investigational product appeared well tolerated and no safety concerns have been identified with the administration of SRT2104 in healthy volunteers at doses up to 3.0 g per day for 7 consecutive days in the fasted state and up to 2.0 g per day for 7 days in the fed state.

Completed and Reported Trials

The first administration of SRT2104 to humans was performed as part of clinical study SRT-2104-001, a randomized, placebo-controlled, single-blind, multiple-dose, dose-escalation study (EudraCT Number: 2007-007598-22). A total of 28 healthy male volunteers received SRT2104 in the form of a liquid suspension as both a single dose and during a separate multiple dose period (once per day for 7 days) at the following dose levels: 30, 100, 250, 500, 1000, 2000, and 3000 mg/day (4 volunteers at each dose level). SRT2104 was well-tolerated following both the single- and multiple-dose periods at all dose levels investigated and all adverse events (AEs) recorded were either mild or moderate in severity and were predominantly gastrointestinal in nature. Treatment-emergent AEs observed in more than one subject were flatulence, headache, nausea, and diarrhea. All AEs were short in duration and resolved

without sequelae. No dose-related AEs were identified. There were no serious adverse events. The pharmacokinetic parameters $AUC_{(0-t)}$ and C_{max} exhibited dose proportionality over the dose range of 30 to 1000 mg/day in healthy volunteers. At doses greater than 1000 mg /day increases in $AUC_{(0-t)}$ and C_{max} were less than proportional to the increase in dose. The terminal elimination half-life (t½) ranged from 8 to 24 hours and was not affected by dose. There was no evidence to suggest saturation of any elimination pathways over the dose range investigated (30 to 3000 mg /day).

A second administration of SRT2104 to human volunteers was performed as part of a single-center, combined intravenous (IV) and oral dosing study to evaluate the PK and absolute bioavailability of SRT2104 (Clinical Protocol SRT-2104-002; EudraCT Number 2008-006283-10). SRT2104 was administered as a single dose of 250 mg administered as an oral suspension and an IV microdose of 100 µg ^{14}C-SRT2104 to eight healthy male subjects. SRT2104 was well tolerated by all subjects. No serious adverse events (SAEs) were recorded. The AEs assessed as related to SRT2104 were aching at infusion site and paresthesia. All AEs were of mild severity and resolved without sequelae. The maximal drug concentrations generally occurred at the end of the 15-minute IV infusion. The terminal elimination half-life for total radioactivity after intravenous dosing was similar to that for the parent drug. The mean $t_{½}$ for ^{14}C-SRT2104 was 23.7 ± 9.37 hours following a 15-minute IV infusion of approximately 100 µg ^{14}C-SRT2104 and mean $t_{½}$ for SRT2104 was 25.5 ± 6.45 hours following oral administration of 250 mg SRT2104. Absolute bioavailability of SRT2104, when dosed as a 250 mg oral suspension in a fasted state, was approximately 14%.

A third clinical study was conducted to assess the effect of food and gender on the PK of SRT2104 (Clinical Protocol SRT-2104-004; EudraCT Number: 2008-007364-41). This Phase I, randomized, open-label study enrolled 10 male and 10 female healthy volunteers to characterize the PK profile of a single 500 mg dose of SRT2104 when administered as an oral suspension and as a capsule formulation in both the fed and fasted states. SRT2104 was well tolerated at the dose level tested. The absorption of SRT2104 administered as capsules and oral suspension was significantly increased when administered to subjects in the fed versus fasted state. There was a very slight decrease in absorption when SRT2104 was administered as a capsule. No gender effects were noted.

Clinical Protocol SRT-2104-009 (EudraCT Number: 2009-010829-39) was a prospective, randomized study to assess the safety and PK of SRT2104 in healthy male volunteers. Ten male subjects were randomized to receive 2000 mg SRT2104 or placebo capsules under fed conditions on eight occasions

during the study, once as a single dose and once per day for seven consecutive days. SRT2104 was considered to be safe and well tolerated at the dose levels tested. The mean $AUC_{(0\text{-}\tau)}$ following once daily dosing for seven days was found to be increased when compared with the mean $AUC_{(0\text{-}\infty)}$ following single dose administration, providing evidence for accumulation of SRT2104 following once daily dosing at 2000 mg for seven days. Evaluation of the pre-dose plasma SRT2104 concentrations during the multiple dose period suggested that steady state had likely not been achieved by all subjects at the end of the 7-day dosing interval. However, review of the individual subject plasma concentrations over the multiple dose period indicated that some subjects appeared to have reached steady state plasma SRT2104 concentrations within seven days of daily dosing.

Following 7 days of dosing of 2 g SRT2104 to fed volunteers the mean C_{max} was 1,874 ng/ml and the mean AUC was 13,997 ng.h/mL. The safety intervals from the 90 day study are 0.50-1.37 ng/mL based on C_{max} and 0.93-3.40 ng.hr/mL based on AUC. It should be pointed out that there were no adverse findings at the highest doses tested in the 90 day studies, so these safety intervals are likely to be an under-estimate of the true values.

Clinical Protocol SRT-2104-008 (EudraCT Number 2009-010620-25) was conducted to assess the pharmacodynamic effect of single oral doses of SRT2104 and prednisolone as measured by levels of *ex vivo* LPS-induced TNF-α production in whole blood of healthy adult subjects. This was a prospective, single center, non-therapeutic, randomized, double-blind study. Twenty male subjects were enrolled in the study and randomized to receive single doses of SRT2104 (250, 500, 1000, and 2000 mg) and/or placebo on 5 separate occasions followed by a single 30 mg dose of prednisolone on the sixth dosing occasion. Although C_{max} increased with SRT2104 dose, the relationship between dose and C_{max} was less than proportional. Median T_{max} did not appear to vary significantly according to dose.

Ongoing Trials

Clinical Protocol SRT-2104-005 (EudraCT Number: 2009-010720-26) is a randomized, placebo-controlled, double-blind, multiple-dose, inpatient/outpatient study to assess the safety and PK of SRT2104 in male and female subjects with type 2 diabetes on an existing, stable, background metformin therapy. Approximately 225 subjects will be enrolled in this study. Subjects will be evenly randomized to receive SRT2104 or placebo in one of five cohorts: placebo (A); 250 mg/day SRT2104 (B); 500 mg/day SRT2104 (C); 1000 mg/day SRT2104 (D); or 2000 mg/day SRT2104 (E). Subjects will be dosed once a day for 28 consecutive days, approximately 15 minutes following the consumption of a standardized

meal. Subjects will remain on a fixed dose of investigational product for all dosing days in the study. As of December 31, 2009, 51 subjects had been randomized into the study, 17 subjects had completed the study, 2 subjects had withdrawn (due to voluntary consent withdrawal), and 13 subjects had experienced treatment-emergent adverse events. A review of blinded safety data showed the most commonly reported adverse events were dyspepsia and hyperglycemia, each of which was reported twice and by different subjects.

Two additional studies of SRT2104 are currently ongoing. Clinical Protocol SRT-2104-007 (EudraCT Number 2009-011918-21) is a randomized, placebo-controlled, double-blind, study to assess the safety, tolerability and pharmacokinetics of oral SRT2104 capsules administered to healthy elderly subjects for 28 days. Twenty-four subjects will be randomized to receive one of the following three treatments: SRT2104 500 mg/day, SRT2104 2000 mg/day or placebo. This study is currently enrolling as is Clinical Protocol SRT-2104-010 (EudraCT Number 2009-011918-21).

Protocol SRT-2104-010 is being conducted to determine if single or multiple doses of SRT2104 attenuate the inflammatory response in normal healthy male subjects after exposure to low-dose endotoxin. Twenty-four healthy male subjects will be randomized to receive single or multiple doses of 2000 mg of SRT2104 and/or placebo for 7 days. Study endpoints include safety, PK and clinical signs and symptoms and laboratory parameters for inflammation.

3 STUDY RATIONALE

A direct role for SIRT1 in promoting keratinocyte differentiation was elucidated [Blander, 2004] and is supportive of earlier findings that resveratrol, a natural plant-derived polyphenol that has been shown to activate SIRT1, inhibited the proliferation of human keratinocytes in vitro [Holian, 2001] and suppressed angiogenesis [Brakenhielm, 2001]. Taken together with the finding that SIRT1 activators are potent inhibitors of cytokine production, including TNF-α [Smith, 2009], the therapeutic potential for SIRT1 activators in the treatment of psoriasis will be explored as part of this clinical protocol. We hypothesize that SIRT1 activators may demonstrate anti-psoriatic action by promoting keratinocyte differentiation, reducing inflammation and/or inhibiting angiogenesis.

4 STUDY OBJECTIVES

4.1 Primary

1. To assess the effects of 250 mg, 500 mg, and 1000 mg SRT2104 on clinical activity in subjects with moderate to severe plaque-type psoriasis based on histological assessment of skin biopsies after 12 weeks of exposure
2. To assess the safety and tolerability of multiple doses of SRT2104 in subjects with moderate to severe plaque-type psoriasis

4.2 Secondary

1. To assess the effects of SRT2104 on the Psoriasis Area and Severity Index (PASI) in subjects with moderate to severe plaque-type psoriasis after 4, 8, and 12 weeks of exposure
2. To assess the effects of SRT2104 on the Physician's Global Assessment (PGA) score in subjects with moderate to severe plaque-type psoriasis after 4, 8, and 12 weeks of exposure
3. To determine the pharmacokinetics of 84 days of dosing with 250 mg, 500 mg, and 1000 mg SRT2104 in the fed state in subjects with moderate to severe plaque-type psoriasis
4. To assess the pharmacodynamic effects of SRT2104 in subjects with moderate to severe plaque-type psoriasis

4.3 Exploratory

1. To characterize expression patterns of select genes and proteins hypothesized to be involved in psoriasis pathophysiology and sirtuin pathways and to evaluate the relationship between these biomarkers and investigational product pharmacokinetics and/or clinical activity
2. To assess the effects of SRT2104 on sense of well-being in subjects with moderate to severe plaque-type psoriasis after 4, 8, and 12 weeks of exposure using the Patient Health Questionnaire-9 (PHQ-9) and the Hospital Anxiety and Depression Scale (HADS)
3. To assess the effects of SRT2104 on health-related quality of life in subjects with moderate to severe plaque-type psoriasis after 12 weeks of exposure using the Koo-Menter Psoriasis Instrument, PQOL-12.

5 INVESTIGATIONAL PLAN

5.1 Overall Study Design

Three cohorts of ten subjects each will be enrolled. Subjects within each cohort will be randomized 4:1 to receive SRT2104 at one of three escalating doses (250, 500, or 1000 mg/day) or placebo. Each cohort of subjects will be dosed sequentially. Dosing in the second and third cohort will not commence until subjects in the previous cohort have completed at least 28 days of dosing, and a review of safety parameters (physical examination findings, vital signs, electrocardiograms, adverse events and laboratory values) has been completed by an Internal Safety Review Committee (ISRC).

Subjects will receive either SRT2104 or matching placebo once daily for 84 days with activity assessments at baseline and after 28, 56 and 84 days of dosing. Safety will be assessed on an ongoing basis. The study consists of a Screening Visit, 7 clinic visits during the dosing period and a follow-up visit.

Potential subjects will sign the IRB-approved informed consent form at the Screening Visit, and will undergo screening assessments to verify eligibility for the study. If eligible and willing to participate, subjects will return to the clinic within 21 days of the Screening Visit to participate in the dosing phase of the study.

For the dosing phase of the study, starting on Day 1, subjects will be randomized on approximately Day -6 to receive either 250 mg, 500 mg, or 1000 mg of SRT2104 or placebo once daily for 84 consecutive days. PK sampling will occur on Days 28, 56 and 84.

5.2 Selection of Study Population

5.2.1 Number of Subjects

A sample size of 8 evaluable subjects per active treatment group and 6 evaluable subjects in the merged placebo group is based on feasibility. Subjects who withdraw from the study prior to 8 weeks of treatment may or may not be replaced (see Section 5.2.2).

5.2.2 Replacement of Subjects

Subjects who withdraw from the study prior to completing 8 weeks of treatment may be replaced.

5.2.3 Inclusion Criteria

A subject will be eligible for inclusion in this study only if all of the following Screening and baseline PASI criteria are met:

1. Able and willing to provide written informed consent to participate in the study

2. Be male or female aged 18 to 80 years (inclusive)
3. Have a diagnosis of clinically confirmed, stable (without recent documented flare within 30 days prior to the Screening Visit), plaque-type psoriasis for at least 6 months involving ≥10% of body surface area
4. Have a baseline PASI of ≥10
5. Be a candidate for systemic psoriasis therapy, in the opinion of the investigator
6. If a female subject of child-bearing potential, be willing to use reliable contraception (see Section 5.15) for the duration of the study, through the 30 day safety follow up visit (a female of child-bearing potential is defined as any female, regardless of her age with functioning ovaries and no documented impairment of oviductal or uterine function that would cause sterility. Females with oligomenorrhea or who are perimenopausal, and young females who have begun to menstruate are considered to be of child-bearing potential)
7. Be willing and able to comply with the protocol for the duration of the study.

5.2.4 Exclusion Criteria

A subject will not be eligible for inclusion in this study if any of the following criteria are met at

Screening:

1. Has received systemic non-biologic psoriasis therapy or PUVA phototherapy within 4 weeks prior to the Screening Visit, or had topical psoriasis treatment or UVB phototherapy within 2 weeks prior to the Screening Visit
2. Has received previous treatment with biologic agents within 5 drug half-lives (or within 3 months if half-life is unknown) prior to the first dose of SRT2104
3. Has received a live vaccination within 4 weeks prior to the Screening Visit or intends to have a live vaccination during the course of the study
4. Use of any other non-psoriatic prescription drug therapy, with the exception of any prescription medication administered at a stable dose for at least 6 weeks prior to the Screening Visit; however, the administration of proton pump inhibitors during the study dosing period is prohibited
5. Use of any dietary or herbal supplements, with the exception of those administered at a stable dose for at least 6 weeks prior to the Screening Visit
6. Has received any investigational drug or experimental procedure within 30 days prior to the first dose of SRT2104
7. Has an active infection (e.g., sepsis, pneumonia, abscess, etc.) or be at high risk of developing an infection, in the opinion of the investigator, prior to the first dose of SRT2104
8. Has a history of a positive tuberculosis test or a positive tuberculosis test at the Screening Visit that cannot be attributed to a prior BCG inoculation
9. Has a positive pre-study Hepatitis B surface antigen or positive Hepatitis C antibody result within 3 months of the Screening Visit
10. Has a positive test for HIV antibody

11. Has an abnormal chest x-ray at the Screening Visit which, in the opinion of the investigator, would preclude entry into the trial
12. Has a 12-lead electrocardiogram (ECG) with changes considered to be clinically significant on medical review including prolonged QTc intervals as defined below:
    - QTcB ≥450 msec (based on single or average QTc value of triplicate ECGs obtained over a brief period)
    - QTcB ≥480 msec in subjects with Bundle Branch Block
13. Has renal or liver impairment, defined as:
    - Serum creatinine level of ≥ 1.4 mg/dL for females and ≥ 1.5 mg/dL for males
    - AST and ALT ≥ 2xULN or
    - Alkaline phosphatase and bilirubin > 1.5xULN (an isolated bilirubin >1.5xULN is acceptable if bilirubin is fractionated and direct bilirubin is <35%)
14. Has active neoplastic disease or history of neoplastic disease within 5 years of study entry (except for basal or squamous cell carcinoma of the skin, or carcinoma in situ which have been definitively treated with standard of care approaches)
15. Is pregnant or breast-feeding. Confirmation that a female subject is not pregnant must be established by negative pregnancy tests at Screening and Day 1
16. Has a significant history of alcoholism or drug/chemical abuse, or consumes more than 3 standard units/day of alcohol. A standard unit of alcohol is defined as 250 mL of beer, 25 mL of spirit, or 125 mL of wine
17. History of sensitivity to any of the study medications, or components thereof, or a history of drug or other allergy that, in the opinion of the investigator or medical monitor, contraindicates their participation
18. Has an acute or chronic illness which, in the opinion of the investigator, could pose a threat or harm to the subject.

5.2.5 Definition of Treatment Failure

A subject who experiences a treatment failure is anyone who demonstrates a score of "no improvement" based on the Krueger criteria defined in Section 6.4, or any subject who is prematurely withdrawn from the study due to lack of efficacy as judged by the investigator.

5.2.6 Removal of Subjects from the Study

Subjects will be informed that they have the right to withdraw from the study at any time for any reason, without prejudice to their medical care. The investigator can withdraw subjects from the study for any of the following reasons:

- QT interval changes as defined in Section 7.1.4
- Liver event (see Section 7.1.5)

- Treatment failure
- Subject request
- Failure to return for follow-up
- Administrative reasons
- General non-compliance with the protocol
- Positive pregnancy test result

The investigator also reserves the right to withdraw subjects in the interest of subject safety and welfare. If a subject is withdrawn from the study, the subject must complete Day 84 assessments at the time of discontinuation and return to the clinic approximately 30 days after the last dose to conduct a safety and activity (PASI and PGA) evaluation.

The sponsor reserves the right to terminate the study in the interest of subject safety as defined in Section 5.11. The sponsor also reserves the right to terminate the study at any time for administrative reasons.

5.3 Selection of Doses in the Study

Three different doses of SRT2104 were selected for study in this protocol, 250 mg/day, 500 mg/day, and 1000 mg/day. These doses should be distinct enough to give different systemic exposures. Comparing three different doses may allow establishment of a pharmacokinetic/pharmacodynamic relationship for SRT2104 and will assist in understanding whether a dose effect exists with regard to the psoriatic efficacy parameters being tested. This will provide valuable information in choosing doses for future trials.

In prior human clinical trials, subjects have received doses of SRT2104 as high as 3000 mg/day with good tolerability. Pharmacokinetic data demonstrated dose-related increases in exposure up to 2000 mg/day, but not beyond. No *in vivo* pharmacodynamic parameters were collected in the normal volunteer studies, thus no human pharmacokinetic/pharmacodynamic relationship has been established to date.

In preclinical testing, SRT2104 has been shown to positively impact a variety of animal models, including those of both metabolic and inflammatory disorders, at daily doses ranging from 10 - 300 mg/kg/day. The most consistent doses associated with good efficacy results in these models range from 100 - 200 mg/kg/day. Adjusting for body surface area between human and mouse, these equilibrate to potential pharmacologically active doses in man of 500 mg to 1000 mg, respectively.

From a safety perspective, the 1000 mg/day dose, the highest dose to be tested, is supported by the pre-clinical safety toxicology package described in Section 2.3 and by the cumulative human safety experience gathered to date. From the pre-clinical 90 day toxicology studies, the safety intervals are 1.3-3.6 based on C_{max} and 1.9-6.8 based on AUC. There were no adverse findings at the highest doses tested in the 90 day studies, so these safety intervals may be an under-estimate of the true values. The most notable finding in the rat and dog toxicity studies has been a small, reversible increase in serum bilirubin. Bilirubin levels will be closely monitored in the proposed clinical trial. The entry criteria are written to exclude subjects with elevated bilirubin levels. Subjects developing elevated bilirubin values that meet the criteria for stopping investigational product will be withdrawn from the study (see Section 7.1.5, Liver Chemistry Stopping Rules and Follow-Up). In the completed and ongoing clinical trials with SRT2104, there have been no serious adverse events reported to date. In addition, no specific safety signals have been identified at this time.

5.4 Identity of Investigational Product

SRT2104 drug substance is a new chemical entity which is supplied as a fine, yellowish/amber powder. The SRT2104 investigational product is prepared by packing 250 mg of micronized SRT2104 powder with no additional additives into a size 00 opaque, hard gelatin capsule, packaged as one, two or four capsules in dosing bottles.

For the matching placebo product, the SRT2104 drug substance will be replaced by microcrystalline cellulose (Avicel® PH 105) to match the SRT2104 investigational product. All subjects will be provided with one dosing bottle per day that contains one, two or four capsules for oral ingestion as described in Section 5.5.

Prior to being dispensed, the investigational product should be stored at 15 – 25 ^{o}C and protected from light. Subjects will be instructed to store investigational product at room temperature away from direct light.

5.5 Treatments to be Administered

Investigational product will be dispensed only to eligible subjects under the supervision of the investigator or identified sub-investigator(s). A trained investigative site member will administer the investigational product to subjects when they are in the clinic, where applicable.

As shown in study SRT-2104-004, the administration of SRT2104 with food increased exposure levels and reduced variability in exposure levels in humans. For this reason, subjects participating in this study

are required to take SRT2104 approximately 15 minutes following the consumption of food. Investigational product should be administered with approximately 250 to 500 cc of water at approximately the same time every dosing day. Subjects should refrain from eating or drinking anything other than water for 1 hour after dosing.

Dietary recommendations for the meal that precedes dosing will be included in the study reference manual and provided to the study subjects by the clinical site staff.

5.6 Method of Assigning Subjects to Treatment Groups

Subjects will be randomized to receive 250 mg, 500 mg, or 1000 mg of SRT2104 or placebo in a 4:1 fashion. Treatments will be administered as follows for the duration of the study:

- Arm 1: 250 mg/day administered as one 250 mg capsule (active or placebo)
- Arm 2: 500 mg/day administered as two 250 mg capsules (active or placebo)
- Arm 3: 1000 mg/day administered as four 250 mg capsules (active or placebo)

5.7 Individuals Who Will Be Unblinded to Treatment Assignments

Designated individuals of Sirtris' Pharmaceutics Research Department will be unblinded for purposes of assigning the treatment assignments according to a randomization schema that will be retained in a secure location with limited access.

In addition, selected personnel at the bioanalytical laboratory will be unblinded to treatment assignments for purposes of data analysis.

Members of the ISRC will be unblinded for purposes of conducting the cohort safety assessments.

5.8 Unblinding Procedures

Procedures for obtaining unblinded treatment information will be provided to the clinical sites in the study operations manual.

If it is medically imperative to know the treatment that a subject is receiving, the investigator shall, prior to requesting the treatment information, make every attempt to contact the medical monitor for the study to discuss the rationale for breaking the blind. In situations where the investigator is unable to contact the medical monitor as described above (e.g., a medical emergency on the part of the subject), the investigator must contact the medical monitor within 24 hours after the code break to inform him/her of the rationale for the code break.

The investigator must provide a written narrative describing the event(s) that led to the code break to the medical monitor within 48 hours following the code break. In addition, the investigator must record the date of the code break and the reasons for breaking the blind in the CRF and in the subject's medical records.

5.9 Duration of Treatment

All subjects enrolled in the study will receive SRT2104 or placebo once daily for up to 84 days during the study period.

5.10 Internal Safety Review Committee

An ISRC will be utilized during the conduct of this study to assess safety and advise on dose escalation as described in Section 5.11. The membership of the ISRC and the plan for the safety data review are described in detail in the ISRC charter. A copy of the ISRC charter is available upon request.

5.11 Safety Review and Dose Escalation or Cessation

Subject safety will be monitored on an ongoing basis.

Dose escalation will be dependent upon the review of the unblinded safety data by the ISRC. All safety data generated for all subjects in the 250 mg and 500 mg cohorts who have completed at least 28 days of dosing will be reviewed by the ISRC. In the event of clinically significant adverse events deemed to be of sufficient severity to pause dosing (refer to criteria in Section 5.11.1), a full analysis of all safety data will be conducted before continuing with a given dose or with dose escalation.

When the last subject in the applicable cohort has completed 28 days of dosing, the data for each subject will be manually entered into an electronic data capture system. The laboratory data will be transferred electronically into the database. The safety data will then be listed and presented to the ISRC for review. If the safety data is acceptable, any subjects still active in the current cohort will continue dosing, and the ISRC will authorize the initiation of dosing to the next cohort of subjects at the higher dose level. This will be repeated until the first 2 cohorts have completed at least 28 days of dosing.

5.11.1 Criteria for discontinuing dosing/dose escalation

In the event that there are SAEs or severe AEs reported in a cohort in which a possible relationship to investigational product cannot be fully excluded, one of the following procedures will be applied:

- If one subject receiving SRT2104 in a cohort experiences a severe AE or an SAE, but the other subjects receiving that same dose are tolerant of that dose, the ISRC and Sponsor will determine

whether the nature, severity, or the number of AEs permit continuing the dosing cohort, or if dosing and/or dose escalation will stop.

- If at least two subjects receiving SRT2104 in a cohort experience a severe AE or an SAE, but similar AEs are recorded for subjects on placebo, the ISRC and Sponsor will determine whether the nature, severity, or the number of AEs permit continuing the dosing cohort, or if dosing and/or dose escalation will stop.
- If at least two subjects receiving SRT2104 in a cohort experience a severe AE or an SAE, and no severe AEs or SAEs are seen in placebo subjects, the dosing of new subjects and/or dose escalation will be halted pending full review by the GSK Global Safety Board.

5.12 Prior Treatments

See the exclusion criteria (Section 5.2.4) for details regarding medications that are prohibited prior to entry into the study.

5.13 Proscribed Medications

In addition to the restrictions described in Section 5.2.4, no other concomitant medications, dietary supplements, or herbal products are permitted for the duration of this trial except as described in Section 5.14.

5.14 Permitted Medications

The following concomitant medications are permitted during the study:

- Non-prescription medications administered to treat an AE (e.g., acetaminophen, or non-steroidal anti-inflammatory agents taken for headache)

  NOTE: Over-the-counter antacids should not be taken within four hours of investigational product administration as they may interfere with the absorption of investigational product.

- Topical Class 6 and/or 7 "rescue" corticosteroid treatment for psoriasis flares may be used, but use must be limited to the face, axillae and groin regions. See Section 13 for a list of permitted topical corticosteroid treatments.

  NOTE: Topical treatments should not be applied to other areas and in particular the plaque being assessed for efficacy, at or near the biopsy site

- Any chronically prescribed non-psoriatic medication or herbal or dietary supplements administered at a stable dose for at least 6 weeks prior to enrollment with the exception of proton pump inhibitors. Potential subjects taking proton pump inhibitors at the time of the Screening Visit must be willing and able to discontinue these medications prior to taking investigational product and for the duration of the dosing period.
- If the investigator desires to initiate therapy with a prescription medication (e.g., in the event of a newly diagnosed medical condition) during the dosing period, s/he should contact the Medical Monitor to discuss the new therapy prior to initiating it.

All medications administered during the study must be recorded in the subject's CRF and in the source documents.

5.15 Contraceptive Use

All female subjects of child-bearing potential must use an adequate form of contraception for the duration of the trial (from the Screening Visit through Day 114). Adequate forms of contraception are defined as:

- Abstinence
- Oral Contraceptive, either combined or progestogen alone
- Injectable progestogen
- Implants of levonorgestrel
- Estrogenic vaginal ring
- Percutaneous contraceptive patches
- Intrauterine device or intrauterine system
- Male partner sterilization (vasectomy with documentation of azoospermia) prior to the female subject's entry into the study, and this male is the sole partner for that subject ("documentation" refers to the outcome of the investigator's/designee's medical examination of the subject or review of the subject's medical history for study eligibility, as obtained via a verbal interview with the subject or from the subject's medical records)
- Double barrier method: condom and an occlusive cap (diaphragm or cervical/vault caps) with a vaginal spermicidal agent (foam/gel/film/cream/suppository)

5.16 Treatment Compliance

Compliance with the investigational product dosing regimen will be assessed by trained study personnel. Subjects are required to bring all study medication (both empty and full containers) to study visits. Study medication containers will be reviewed by the site staff at each study visit to verify treatment compliance.

Subjects who do not comply with the investigational product dosing regimen and who consequently miss either three consecutive doses or six individual doses of investigational product at any time point during the study may be withdrawn from the study. The Investigator must call the medical monitor to discuss the disposition of subjects who miss doses as described above.

5.17 Missed Doses of Investigational Product

Subjects who inadvertently miss a dose of investigational product should skip that dose and recommence dosing on the next dosing day.

5.18 Schedule of Events

A detailed visit-by-visit schedule of study procedures is provided in Section 1.1. Prior to engaging in any study procedure, each subject must sign and date an IRB-approved informed consent form.

6 STUDY PROCEDURES

The timepoints for all study procedures are reflected in the Schedule of Events (see Section 1.1). Visit windows are as follows: a plus/minus 4-day window will be applied to Visits 3 through 7; a 1-day plus/minus window will be applied to Visit 8 (Day 84). The date of the visit will be recorded in the CRF.

Descriptions of the required study procedures are provided below:

Physical Examination

A complete physical examination should be performed at Screening. A symptom-driven, directed physical examination will be performed at other timepoints as defined in the schedule of events. Body systems that will be included are the following: general appearance, musculoskeletal, skin and mucosa, head and neck, lymphatic, respiratory, breast, gastrointestinal, cardiovascular, extremities, neurological, psychological, eyes, ears, nose and throat. Other body systems may be examined as needed, at the discretion of the investigator.

Body Weight/Height

Height is measured once at the Screening visit. Body weight will be measured at all other clinic visits. The clinical staff will be instructed to use calibrated scales to measure subjects' body weight.

Vital Signs

Vital sign assessments will include measurements of resting pulse rate, blood pressure, respiration rate, and temperature.

Electrocardiogram

A 12-lead ECG will be performed in the rested state. The subject should be lying comfortably in the supine position with ECG leads on for at least 5 minutes prior to the ECG recording. The ECGs will be read locally by the clinical site. The ECG will include the assessment of PR (PQ), QRS, QT, and QTc intervals and heart rate. Identification of any conduction abnormalities will be recorded in the CRF. If a subject's QTc intervals are prolonged, then the ECG should be done in triplicate with results reported as an average of the three ECGs. Copies of the ECG graphs and available reports will be collected by the study sponsor at the end of the study.

Prolonged QTc intervals will be managed according to the QTc withdrawal criteria described in Section 7.1.4.

<u>Pregnancy Testing</u>

Serum pregnancy testing will be performed at the Screening Visit; urine pregnancy tests will be performed at all other visits where a pregnancy test is performed (see Section 1.1).

<u>Tuberculosis Test</u>

A tuberculosis test will be performed at the Screening Visit. A positive test result that cannot be attributed to a prior BCG inoculation will disqualify the subject. (see Section 5.2.4)

<u>Blood and Urine Sample Collection for Clinical Laboratory Testing</u>

Blood and urine samples will be collected as outlined in the Schedule of Events (see Section 1.1) for clinical laboratory safety testing, serology and biomarker analyses. See Section 6.3 for a complete listing of blood and urine parameters.

Sample collection, processing, and shipping instructions for samples that will not be analyzed in the local laboratory at the clinical site will be provided in the study operations manual.

<u>Pharmacokinetics</u>

Approximately 20 mL of blood will be collected throughout the duration of the trial for PK analyses. Samples will be collected on Days 28, 56 and 84 using sparse sampling techniques as described in Section 6.2. Analyses may include metabolite profiling in a subset of samples. Collected samples will be transferred for analysis to Simbec Research Ltd., South Wales, United Kingdom.

Additional sample collection, processing, and shipping instructions will be provided in the study operations manual.

<u>Pharmacodynamics</u>

Biomarkers of psoriatic disease activity and/or sirtuin pathway activation will be analyzed in blood samples collected for this purpose on Days 1, 28, 56 and 84 and may include, but may not be limited to, hsCRP and FGF21.

<u>Pharmacogenetics</u>

A single blood sample will be obtained on Day 1 for potential pharmacogenetic analysis. The sample is labelled (or "coded") with a study-specific number that can be traced or linked back to the subject by the investigator or site staff. Coded samples do not carry personal identifiers such as name or social security number. A full description of the pharmacogenetic assessment is included in Appendix 2. Subject participation in the pharmacogenetic portion of the study is voluntary and refusal to participate will not preclude participation in the clinical study.

Photography

Subjects who provide consent to photography procedures will have full body photographs taken from the neck down at timepoints outlined in the Schedule of Events (see Section 1.1). Identifying marks such as tattoos that might reveal the identity of a subject will be covered.

Skin Biopsy

A skin biopsy will be collected on Days 1 and 84 and transferred to the Rockefeller University, New York, New York, USA, for evaluation by a central reader. Biopsy tissue analyses are described in detail in Section 6.4.

Skin biopsy collection, processing, and shipping instructions will be provided in the study operations manual.

Quality of Life Assessments

Some authors have noted that the psychosocial consequences of psoriasis can be as disruptive to the individual's life as the physical aspects of the illness itself [Fortune DG, 1997]. As part of an effort to explore whether improvements in the psychosocial consequences of psoriasis represent potential independent targets for treatment with SRT2104, the present study includes the three patient-reported outcome instruments described below.

The Patient Health Questionnaire 9 (PHQ-9) is a 9-item, validated self-rating instrument that assesses eight core symptoms of depression in addition to an item that assesses functioning. The PHQ-9 has been validated as a diagnostic instrument for major depression, and it also provides an assessment of the severity of depression [Kroenke K, 2001].

The Hospital Anxiety and Depression Scale (HADS) is a 14-item, validated self-rating instrument of symptoms of anxiety and depression [Mykletun A, 2001]. It is intended to provide a cross-sectional assessment of anxiety and depressive symptoms whether or not subjects meet formal criteria for anxiety

and depressive disorders. As such, the HADS could provide a signal of efficacy in a broad-based population of psoriasis sufferers.

The 12-item Psoriasis Quality of Life questionnaire (PQOL-12) is a brief, validated instrument derived from a 41-item instrument covering both psychosocial and physical domains [Feldman SR, 2005; Koo J, 2002]. The focus of the PQOL-12 on patient reports of psychosocial and physical effects specifically related to psoriasis reflects the growing emphasis among dermatologists on the major impact of the illness on patients' quality of life.

Adverse Event Monitoring

AE assessment (including SAEs) will be assessed on an ongoing basis throughout the study starting from the time of first dose. All SAEs/SUSARs should be monitored until they are resolved or clearly determined to be due to a subject’s stable or chronic condition or intercurrent illness(es). Definitions, documentation, and reporting of AEs are described in detail in Section 7.

Concomitant Medications

Concomitant medication usage will be assessed throughout the study. See Section 5.13 for a description of permitted and restricted concomitant medications.

6.1 Study Procedures

6.1.1 Visit 1 (Screening)

Potential subjects will be given an opportunity to have any questions about the study or their participation in it answered by the principal investigator or his designate. Prior to engaging in any study procedure, each potential subject must sign and date an IRB-approved informed consent form. When the consent form is signed, each subject will be assigned a unique screening number and screening procedures will be performed.

6.1.2 Visit 2

Those subjects meeting the study entry criteria who agree to participate in the study will return to the clinical site on Day 1. The investigator may, at his/her discretion choose to conduct a portion of the Day 1 visit procedures (exclusive of investigational product administration) on Day -1. Study procedures including safety, activity (PASI and PGA) and QOL assessments will be performed as outlined in the Schedule of Events (Section 1.1). Blood samples for biomarker analysis, pharmacogenetics and skin biopsies will be obtained. In addition, a photograph will be taken to document the body surface area

affected by psoriatic lesions. Subjects will be randomized to a treatment group approximately 6 days prior to this visit to allow sufficient time for delivery of investigational product. The first dose of study medication will be administered in the clinic following consumption of food. Prior to leaving the clinic, subjects will receive a kit containing study medication and will be instructed to continue once daily dosing after eating, and to store the study medication under ambient conditions (between 15 and 25 ºC), protected from direct light for the remainder of the dosing period.

6.1.3 Visits 3, 5 and 7

On Days 14, 42 and 70 subjects will return to the clinic for safety assessments as outlined in the Schedule of Events (Section 1.1).

6.1.4 Visits 4 and 6

On Days 28 and 56 subjects will return to the clinic for safety, activity, well-being and QOL assessments, PK sampling and pregnancy tests as outlined in the Schedule of Events (Section 1.1). A photograph will be taken to document the body surface area affected by psoriatic lesions. Wherever possible, based on scheduling of the subject visits, investigational product will be administered in the clinic to facilitate the accurate recording of dosing time and PK sampling times. Information regarding AEs and concomitant medications will be collected.

6.1.5 Visit 8

On Day 84 subjects will return to the clinic for safety, activity, well-being and QOL assessments, PK sampling and pregnancy tests as outlined in the Schedule of Events (Section 1.1). A photograph will be taken to document the body surface area affected by psoriatic lesions. Skin biopsies and blood samples for biomarker analysis will be obtained. Wherever possible, based on scheduling of the subject visits, investigational product will be administered in the clinic to facilitate the accurate recording of dosing time and PK sampling times. Information regarding AEs and concomitant medications will be collected.

6.1.6 Follow-Up Procedures

On Day 114 subjects will return to the clinic for safety and activity assessments, and pregnancy testing as outlined in the Schedule of Events (Section 1.1).

6.2 Pharmacokinetic Sampling

PK measurements will be required for all subjects at all participating centers. Accurate recording of dosing and sample collection times is critical. The actual dosing and sample collection times must be recorded in the CRF.

A total of five blood samples (6 mL each) will be obtained from each subject over the course of the study for determination of SRT2104 plasma concentrations. The following samples can be taken over the course of Visits 4, 6, and 8. Multiple PK samples can be taken during one visit provided the sampling windows as described below are observed and no 2 samples are separated by less than 1 hour.

- One pre-dose sample will be collected prior to taking investigational product (30 minutes or less before dosing). This sample must be collected on any ONE of the following: Visit 4, 6 or 8. It is recommended that the dose associated with this sample be administered in the clinic.
- A single PK sample will be collected in the time interval of 0.5 to 2 hours post-dose. It is recommended that the dose associated with this sample be administered in the clinic. This sample must be collected on any ONE of the following: Visit 4, 6 or 8.
- A single PK sample will be collected in the time interval of 3 to 6 hours post-dose. This sample must be collected on any ONE of the following: Visit 4, 6 or 8.
- Two PK samples will be collected in the time interval of 6 to 22 hours post-dose. These 2 samples must be collected on any of the following: Visit 4, 6 or 8.

PK samples may be collected at any time during the defined sampling intervals. An effort should be made to ensure that samples are not consistently collected at the same time point within a defined collection interval. This study provides flexible options for scheduling of most PK samples. This flexibility is incorporated to improve subject convenience for sampling at later time points.

6.3 Laboratory Assessments

Safety (e.g., hematology, chemistry, and urinalysis) and screening (e.g., serum β-HCG and serology) samples will be analyzed by a central laboratory. A subset of samples (e.g., PK and FGF21 samples) may be transferred for analysis to Sirtris, GlaxoSmithKline (GSK), or other designated representative working with GSK and/or Sirtris.

The following clinical laboratory parameters will be evaluated at the time points specified in the Schedule of Events (see Section 1.1). Collected samples may be transferred for analysis to Sirtris, GSK, or other designated representatives working with GSK or Sirtris.

Hematology

White blood cell count (WBC)	Complete WBC differential
Hemoglobin	Platelets
Hematocrit	Mean corpuscular volume
Red blood cell count	Mean Corpuscular Hemoglobin Concentration
Red Cell Distribution Width	Mean Corpuscular Hemoglobin

Clinical Chemistry

Sodium	Alanine aminotransferase
Potassium	Aspartate aminotransferase
Chloride	Total, direct, and indirect bilirubin
Blood Urea Nitrogen	Alkaline phosphatase
Serum creatinine	Lactate dehydrogenase
Plasma Glucose	Gamma-glutamyl transferase
Calcium	Amylase
Magnesium	Bicarbonate
Uric acid	Albumin
ProthrombinTime/International Normalized Ratio [1]	Phosphate
Activated Partial Thromboplastin Time [1]	Creatine Kinase

Lipid Profile (Total Cholesterol, Low Density Lipoprotein, High Density Lipoprotein, Free Fatty Acids, Triglycerides)[2]

[1] Coagulation studies will be performed at Screening and on Days 1, 42 and 84 only.

[2] Lipid profiling will be performed in fasting samples at Screening, Days 1, 42 and 84 only.

Pregnancy Monitoring

Serum β-HCG at Screening	Urine testing at other timepoints as defined in the Schedule of Events (Section 1.1)

Urinalysis

Dipstick	Microscopic exam only if dipstick abnormal

Serology

HCV ab	HBsAg
HIV 1 & 2	

Biomarkers*

hsCRP	FGF21

*Biomarker analysis will be performed in fasting samples according to the timepoints displayed in the Schedule of Events (Section 1.1). Additional biomarkers thought to be associated with SIRT1 activation may be assessed.

6.4 Skin Biopsy Evaluation and Scoring

Skin biopsies will be obtained from the same designated plaque on Days 1 and 84. The central reader at Rockefeller University (who will be blinded to treatment assignments) will evaluate the biopsy tissue for general appearance, epidermal thickness, total inflammatory infiltrate, specific cell numbers (including but not limited to CD163+ monocytes, CD11c+ dendritic cells, CD83+and/or CD206+ cells, and CD3+ T-cells). Keratinocyte expression of K-16 and ICAM-l will be measured. RT-PCR will be used to assess for expression of specific genes which may include, but not be limited to, K-16, IL-1β, IL-6, IL-8, IL-10, IL-12, IL-17, IL-22, IL-23, INFγ, TNF-α, iNOS, IL-1R antagonist, PGC-1α, NCoR, NFκβ, FOXO, p300, PPARα, PPAR-delta, and p53. In addition, global changes in gene expression may be assessed using gene micro-array techniques.

Skin biopsies will be assigned an improvement score according to the Krueger criteria defined below:

Improvement Score	Definition
No improvement	defined as no improvement in epidermal thickness keratinocyte differentiation or K16 expression on keratinocytes
Good improvement	defined as reduction in epidermal thickness by at least 30% normalized keratinocyte differentiation but most keratinocytes still express K16
Excellent improvement	defined as reduction in epidermal thickness to normal or almost normal normalized keratinocyte differentiation and absent keratinocyte expression of K16.

6.5 Appropriateness of Measurements

Per the U.S. Food and Drug Administration (FDA) guidance, “Collection of Race and Ethnicity Data in Clinical Trials, September 2005”, demographic data and complete subject medical histories will be

documented for all subjects during screening. Investigational product administration data, including dose interruptions and modifications and the associated reason(s), also will be documented.

AEs and SAEs will be monitored in this study in accordance with International Conference on Harmonization (ICH) Good Clinical Practice (GCP) guidelines to ensure the safety of subjects.

6.6 Data Quality Assurance

Sirtris or its designated representative will conduct a clinical site visit to verify the qualifications of the investigator, inspect clinical site facilities, and inform the investigator of responsibilities and procedures for ensuring adequate and correct study documentation.

The investigator is required to prepare and maintain adequate and accurate case histories designed to record all observations and other data pertinent to the study for each study participant. All information recorded on the CRFs for this study must be consistent with the subject’s source documentation.

During the course of the study, the study monitor will conduct clinical site visits to review protocol compliance, compare CRFs and individual subject’s medical records (source documents), assess investigational product accountability, and ensure that the study is being conducted according to pertinent regulatory requirements. CRFs will be verified with source documentation. The review of medical records will be performed in a manner to ensure that subject confidentiality is maintained. A hardcopy of the final CRFs will be placed in the investigator’s study file and Sirtris’ Trial Master File (TMF).

Instances of missing or uninterpretable data will be discussed with the investigator for resolution. Study data will be entered into a secure, validated data processing system and a backup will be maintained. Any changes to study data will be documented. A quality assurance audit will be performed on the database.

7 ADVERSE EVENTS

The principal investigator and designated study staff are responsible for detecting, documenting, and reporting events that meet the definition of an AE or SAE/SUSARs.

7.1 Definitions

7.1.1 Adverse Event Definition

An **adverse event** (AE) is any untoward medical occurrence in a subject administered a pharmaceutical product, which does not necessarily have a causal relationship with the treatment. An AE can be any unfavorable and unintended sign (e.g., including an abnormal laboratory finding), symptom, or disease temporally associated with the use of the investigational product, whether or not it is considered to be investigational product-related. This includes any newly occurring event or previous condition that has increased in severity or frequency since the administration of investigational product.

7.1.2 Serious Adverse Event Definition

A **serious adverse event** (SAE) is any AE, occurring at any dose and regardless of causality that:

- Results in **death**.
- Is **life-threatening**. Life-threatening means that the subject was at immediate risk of death from the reaction as it occurred, i.e., it does not include a reaction which hypothetically might have caused death had it occurred in a more severe form.
- Requires inpatient **hospitalization or prolongation of existing hospitalization**. Hospitalization admissions and/or surgical operations scheduled to occur during the study period, but planned prior to study entry, are not considered AEs if the illness or disease existed before the subject was enrolled in the trial, provided that it did not deteriorate in an unexpected manner during the study (e.g., surgery performed earlier than planned).
- Results in **persistent or significant disability/incapacity**. Disability is defined as a substantial disruption of a person’s ability to conduct normal life functions.
- Is a **congenital anomaly/birth defect**.

- Is an **important medical event**. An important medical event is an event that may not result in death, be life-threatening, or require hospitalization but may be considered an SAE when, based upon appropriate medical judgment, it may jeopardize the subject and may require medical or surgical intervention to prevent one of the outcomes listed in the definitions for SAEs. Examples of such medical events include allergic bronchospasm requiring intensive treatment in an emergency room or at home, blood dyscrasias or convulsions that do not result in inpatient hospitalization, or the development of drug dependency or drug abuse. See Section 7.1.5 for guidance on liver events that would be considered SAEs.
- Is associated with **liver injury and impaired liver function** defined as:
  - ALT ≥ 3xULN, and
  - total bilirubin ≥ 2xULN or INR > 1.5.

  NOTES: (1) Bilirubin fractionation should be performed if testing is available. If fractionation is unavailable, urinary bilirubin is to be measured via dipstick (a measurement of direct bilirubin, which would suggest liver injury). (2) INR measurement is not required; if measured, the threshold value stated will not apply to subjects receiving anticoagulants. If INR measurement is obtained, the value is to be recorded on the SAE form.
- **Pregnancy complications**: spontaneous abortions in subjects exposed to investigational product must be reported as SAEs.

  NOTE: Any SAE occurring in association with a pregnancy that is brought to the investigator's attention after the subject has completed the study and that is considered by the investigator as possibly related to the investigational product, must be promptly reported to Sirtris.

Clarification should be made between the terms "serious" and "severe" as they ARE NOT synonymous. The term "severe" is often used to describe the intensity (severity) of a specific event (as in mild, moderate, or severe myocardial infarction); the event itself, however, may be of relatively minor medical significance (such as a severe headache). This is NOT the same as "serious," which is based on subject/event outcome or action criteria described above, and is usually associated with events that pose a threat to a subject's life or functioning. A severe AE does not necessarily need to be considered serious. For example, persistent nausea of several hours' duration may be considered severe nausea but not an

SAE. On the other hand, a stroke resulting in only a minor degree of disability may be considered mild, but would be defined as an SAE based on the above noted criteria. Seriousness (not severity) serves as a guide for defining regulatory reporting obligations.

7.1.3 Suspected Unexpected Serious Adverse Reaction Definition

A suspected unexpected serious adverse reaction (SUSAR) is any adverse drug reaction (i.e., any AE that is assessed by the Investigator as associated with [i.e., unlikely, possibly or probably related to] SRT2104, the specificity or severity of which is not consistent with those noted in the current protocol and/or Investigator's Brochure (IB). This refers to any adverse reaction that has not been previously observed (e.g., included in the IB), rather than from the perspective of such an event not being anticipated from the pharmacological properties of the product.

7.1.4 QTc Withdrawal Criteria

A subject that meets the criteria below will be withdrawn from the study.

- QTcB > 500 msec (machine or manual overread). If the subject has bundle branch block then the criterion is QTcB > 530 msec
- Prolongation of QTcB > 60 msec as compared to baseline

These criteria are based on an average QTc value of triplicate ECGs. If an ECG demonstrates a prolonged QT interval, 2 additional ECGs are to be obtained over a brief period and the averaged QTc values of the 3 ECGs is used to determine whether the subject should be discontinued from the study. All ECGs with a machine-read QTcB value of > 500 msec collected during the study (including at Screening) will be manually over-read by a cardiologist to verify the QTcB value.

7.1.5 Liver Chemistry Stopping Rules and Follow-up

Liver Chemistry Stopping Rules

Investigational product will be stopped if any of the following liver chemistry stopping criteria is met:

1. ALT ≥ 3xULN and bilirubin ≥ 2xULN (or ALT ≥ 3xULN and INR > 1.5)

   NOTE: serum bilirubin fractionation should be performed if testing is available. If fractionation is unavailable, urinary bilirubin is to be measured via dipstick (a measurement of direct bilirubin, which would suggest liver injury).

2. ALT ≥ 5xULN
3. ALT ≥ 3xULN if associated with the appearance or worsening of rash or hepatitis symptoms (fatigue, nausea, vomiting, right upper quadrant pain or tenderness, fever, rash or eosinophilia)
4. ALT ≥ 3xULN persists for ≥ 4 weeks
5. ALT ≥ 3xULN and cannot be monitored weekly for 4 weeks
6. Alkaline phosphatase ≥ 3xULN **and** bilirubin ≥ 2xULN.
7. Subjects with ALT ≥ 3xULN **and** < 5xULN **and** bilirubin < 2xULN, who do not exhibit hepatitis symptoms or rash, can continue investigational product as long as they can be monitored weekly for 4 weeks.

Liver Chemistry Follow-up

If any of criteria 1 through 5 above are met, the following actions should be taken:

- **Immediately** withdraw investigational product
- Report the event to Sirtris **within 24 hours** of learning its occurrence
- Complete the SAE reporting form if the event also meets the criteria for an SAE. All events of ALT ≥ 3xULN **and** bilirubin ≥ 2xULN (>35% direct bilirubin) (or ALT ≥ 3xULN **and** INR >1.5, if INR measured; INR measurement is not required and the threshold value stated will not apply to subjects receiving anticoagulants), termed 'Hy's Law', **must be reported as an SAE**.

NOTE: if serum bilirubin fractionation is not immediately available, investigational product should be discontinued if ALT ≥ 3xULN **and** bilirubin ≥ 2xULN. Serum bilirubin fractionation should be performed if testing is available. If testing is unavailable, **record presence of detectable urinary bilirubin on dipstick**, indicating direct bilirubin elevations and suggesting liver injury.

- Perform liver event follow up assessments, and monitor the subject until liver chemistries resolve, stabilize, or return to baseline values as described below
- Withdraw the subject from the **study** (unless further safety follow up is required) after completion of the liver chemistry monitoring as described below

- Do not re-challenge with investigational product.

In addition, for criterion 1:

- Make every reasonable attempt to have subjects return to clinic within **24 hours** for repeat liver chemistries, liver event follow up assessments (see below), and close monitoring
- A specialist or hepatology consultation is recommended
- Monitor subjects twice weekly until liver chemistries (ALT, AST, alkaline phosphatase, bilirubin) resolve, stabilize or return to within baseline values

For criteria 2, 3, 4 and 5:

- Make every reasonable attempt to have subjects return to clinic **within 24-72 hrs** for repeat liver chemistries and liver event follow up assessments (see below)
- Monitor subjects weekly until liver chemistries (ALT, AST, alkaline phosphatase, bilirubin) resolve, stabilize or return to within baseline values; criterion 5 subjects should be monitored as frequently as possible.

For criteria 6 and 7:

- Notify the Sirtris medical monitor within 24 hours of learning of the abnormality to discuss subject safety
- Can continue investigational product
- Must return weekly for repeat liver chemistries (ALT, AST, alkaline phosphatase, bilirubin) until they resolve, stabilize or return to within baseline
- If at any time these subjects meet the liver chemistry stopping criteria, proceed as described above
- If, after 4 weeks of monitoring, ALT < 3xULN and alkaline phosphatase < 3xULN and bilirubin < 2xULN, monitor subjects twice monthly until liver chemistries normalize or return to within baseline values.

For criteria 1-5, make every attempt to carry out the assessments described below:

- Viral hepatitis serology including:
  - Hepatitis A IgM antibody;
  - Hepatitis B surface antigen and Hepatitis B Core Antibody (IgM);
  - Hepatitis C RNA;
  - Cytomegalovirus IgM antibody;
  - Epstein-Barr viral capsid antigen IgM antibody (or if unavailable, obtain heterophile antibody or monospot testing);
  - Hepatitis E IgM antibody (if subject has travelled outside US in past 3 months);
- Blood sample for PK analysis, obtained as soon as possible but no later than 5 days following last dose. Record the date/time of the PK blood sample draw and the date/time of the last dose of investigational product prior to blood sample draw on the CRF. If the date or time of the last dose is unclear, provide the subject's best approximation. If the date/time of the last dose cannot be approximated OR a PK sample cannot be collected in the time period indicated above, do not obtain a PK sample. Instructions for PK sample handling and shipping are in the study operations manual.
- Serum creatine phosphokinase and lactate dehydrogenase
- Fractionate bilirubin, if total bilirubin ≥ 2xULN
- Obtain complete blood count with differential to assess eosinophilia
- Record the appearance or worsening of clinical symptoms of hepatitis, or hypersensitivity, such as fatigue, nausea, vomiting, right upper quadrant pain or tenderness, fever, rash or eosinophilia as relevant on the AE report form
- Record use of concomitant medications, acetaminophen, herbal remedies, other over the counter medications, or putative hepatotoxins, on the concomitant medications report form
- Record alcohol use on the liver event alcohol intake case report form

The following are required for subjects with ALT ≥ 3xULN and bilirubin ≥ 2xULN (>35% direct) but are optional for other abnormal liver chemistries:

- Anti-nuclear antibody, anti-smooth muscle antibody, and Type 1 anti-liver kidney microsomal antibodies
- Liver imaging (ultrasound, magnetic resonance, or computerized tomography) to evaluate liver disease.

7.2 Procedures for Recording and Reporting AEs and SAEs

All AEs spontaneously reported by the subject and/or in response to an open question from study personnel or revealed by observation, physical examination or other diagnostic procedures will be recorded on the appropriate page of the CRF. Any clinically relevant change in laboratory assessments or other clinical findings is considered an AE and must be recorded on the appropriate pages of the CRF. When possible, signs and symptoms indicating a common underlying pathology should be noted as one comprehensive event.

All SAEs and SUSARs that occur during the course of the study, as defined by the protocol, must be reported by the investigator to Sirtris by faxing the SAE/SUSAR form **within 1 working day** from the point in time when the investigator becomes aware of the SAE/SUSAR. In addition, all SAEs/SUSARs, including all deaths, which occur up to and including 30 days after administration of the last dose of investigational product, must be reported to Sirtris within 1 working day. All SAEs/SUSARs and deaths must be reported whether or not considered causally related to the investigational product. The information collected using the SAE/SUSAR form will include a minimum of the following: subject number, a narrative description of the event and an assessment by the investigator as to the intensity of the event and relatedness to investigational product. A sample of the SAE/SUSAR form can be found in the study operations manual. Follow-up information on the SAE/SUSAR may be requested by Sirtris.

SAE/SUSAR Reporting Contact Information:
Sirtris Pharmacovigilance
Sirtris Pharmaceuticals
200 Technology Square
Cambridge, MA 02139 USA
Fax Line: 1 617 679 8499 or
Email: SirtrisPVG@sirtrispharma.com

and to the Medical Monitor:
Eric Jacobson, MD
Chief Medical Officer
Sirtris Pharmaceuticals
200 Technology Square
Cambridge, MA 02139 USA
Direct Line: 1 617 252 6920, Extension 2208

If there are suspected, unexpected, serious adverse drug reactions (SUSARs) associated with the use of the investigational product, Sirtris or its designee will notify the appropriate regulatory agency(ies) and all participating investigators on an expedited basis (7 days for fatal or life-threatening suspected, unexpected, serious adverse drug reactions). Sirtris has delegated the responsibility to promptly notify the Institutional Review Board (IRB)/Independent Ethics Committee (IEC) of all SUSARs involving risk to human subjects in accordance with the rules and regulations of the IRB/IEC to the principal investigator. An unexpected event is one that is not reported in the Investigator's Brochure.

Planned hospital admissions or surgical procedures for an illness or disease that was diagnosed before the subject was enrolled in the study or before investigational product was given, are <u>not</u> to be considered AEs.

For both serious and non-serious AEs, the investigator must determine both the intensity and the relationship of the event to investigational product administration.

Intensity for each AE will be determined by using the National Cancer Institute (NCI) Common Toxicity Criteria (CTC), Version 4.0 as a guideline, wherever possible. Dose-limiting toxicities will be defined as those AEs of Grade 3 or greater that are seen to occur with an evident temporal relationship to SRT2104

dosing. In those cases where the NCI CTC criteria do not apply, intensity should be defined according to the following criteria:

Mild	Awareness of sign or symptom, but easily tolerated
Moderate	Discomfort enough to cause interference with normal daily activities
Severe	Inability to perform normal daily activities
Life Threatening or Disabling	Immediate risk of death from the reaction as it occurred
Death	The event resulted in death

Relationship to investigational product administration will be determined as follows:

Not Related	No relationship between the experience and the administration of investigational product; related to other etiologies, such as concomitant medications or subject’s clinical state.
Unlikely Related	The current state of knowledge indicates that a relationship is unlikely.
Possibly Related	A reaction that follows a plausible temporal sequence from administration of the investigational product and follows a known response pattern to the suspected investigational product. The reaction might have been produced by the subject’s clinical state or other modes of therapy administered to the subject.
Related	A reaction that follows a plausible temporal sequence from administration of the investigational product and follows a known response pattern to the suspected investigational product and can be confirmed with a positive re-challenge test or supporting laboratory data.

7.3 Monitoring of Adverse Events and Period of Observation

AEs, both serious and non-serious, and deaths will be recorded on the CRFs throughout the study from the time of first dose until the final follow-up contact. However, any SAEs assessed as related to study participation (e.g., investigational product, protocol-mandated procedures, invasive tests, or change in existing therapy) will be recorded from the time a subject consents to participate in the study up to and including any follow-up contact. All SAEs should be monitored until they are resolved or are clearly determined to be due to a subject's stable or chronic condition or intercurrent illness(es). Any SAE that occurs at any time after completion of the study including the designated follow-up period, which the investigator considers to be related to study medication, must be reported to Sirtris.

AEs or SAEs requiring therapy must be treated by recognized standards of medical care to protect the health and well-being of the subject. Appropriate resuscitation equipment and medicines must be available to ensure the best possible treatment of an emergency situation.

The outcome of AEs will be rated as:

- Recovered/Resolved;
- Recovering/Resolving;
- Not Recovered/Not Resolved;
- Recovered/Resolved with Sequelae;
- Fatal;
- Unknown.

Any non-serious AE which occurs in the course of the study should be monitored and followed for up to 30 days following the last dose of investigational product.

7.4 Pregnancy Reporting

The investigator will attempt to collect pregnancy information on any female subject who becomes pregnant while participating in this study. The investigator will record pregnancy information on the appropriate form and submit it to Sirtris within 2 weeks of learning of the pregnancy. The outcome of the pregnancy will also be followed. Any premature termination of the pregnancy will be reported.

Information on the status of the mother and child will be forwarded to Sirtris at the time of birth, where applicable. Generally, follow-up of live births will occur at approximately 3-month intervals and will be no longer than 12 months following the estimated delivery date. Pregnancy complications, including spontaneous abortions, and the medical reason(s) for elective terminations must be reported as AEs or SAEs.

8 STATISTICAL PROCEDURES

The primary objectives are (1) to assess the effects of SRT2104 on clinical activity, based on the Krueger criteria, relative to a historical placebo response rate of 5% and (2) to assess safety and tolerability of SRT 2104. For the first objective the analysis will be based on an exposure-response analysis. Previous studies have indicated that the pharmacokinetic exposure is relatively variable. For this reason, the active treatments will be combined and then dichotomized into low and high drug exposure groups. The cut-off point will likely be the midpoint of exposure but the selection of the final cut-point will be dependent on the distribution of exposure. Point estimates and 90% confidence intervals will be constructed for the differences between the proportion of responders, defined as “good” or “excellent” Krueger improvement score, for each of the exposure groups and the historical null placebo response of 5%. No formal hypothesis testing will be performed. The second primary objective will be accomplished by review of individual subject data and descriptive summaries of the safety data. Safety displays will be summarized by treatment group (placebo, 250mg, 500mg, and 1000mg).

Point estimates and 90% confidence intervals will also be constructed for the secondary endpoints of clinical activity (PASI-50, PASI-75, and PGA of “almost clear” or “clear”) for the differences between the proportion of responders for each of the exposure groups and the historical null placebo response of 5%. The clinical activity endpoints will also be summarized by treatment group, as a secondary analysis. All other pharmacodynamic endpoints will summarized by treatment group. Point estimates and corresponding 90% confidence intervals will be constructed for PK comparisons.

8.1 Randomization and Stratification

On entry into each of the three cohorts, the study subjects will be randomized to active SRT2104 (250mg, 500 mg, or 1000 mg) or placebo in a 4:1 fashion using a predetermined randomization schedule. All subjects will receive the assigned, double-blind study medication for 84 days.

8.2 Sample Size

A sample size of 8 evaluable subjects per active treatment group and 6 evaluable placebo subjects in a merged placebo group is based on feasibility.

The following information is provided to estimate the precision of the estimates, assuming SRT2104 histology success rate (an improvement score of “good or excellent” based on the Krueger criteria [see Section 6.4] of .33%) and the exposure data is dichotomized at the midpoint (n=12 for each exposure

group).

True Krueger response rate	Null placebo response rate	Power	Cut-off value for rejecting Ho	Difference between true and null response rate	90% CI for the difference*
.33	.05	.8124	3	.28	(0.1229,0.6084)
				.20	(0.0719, 0.5272)
.33	.10	.5973	4	.23	(0.1230,0.6090)
				.15	(0.0720, 0.5272)

*90% CI is based on exact methods

Note, if hypothesis testing was performed, instead of an estimation approach, based on the twelve subjects: a single sample exact binomial test with a nominal 0.05 one-sided significance level will have 82% power to detect the difference from the between a null placebo response success rate of 5%, given the and a true SRT2104 histology success response rate (an improvement score of "excellent improvement" based on the Krueger criteria [see Section 6.4]) of 33.0%. Subjects who withdraw from the study prior to 8 weeks of treatment may or may not be replaced (see Section 5.2.2).

In addition, with a sample size of 8 for active dose groups, the probability of observing at least one adverse event of a given type will be 72.8% when the probability of such an adverse event is actually 15.0%.

Based on the results of the second cohort (SRT2104 500 mg) the sample size of the last cohort (SRT2104 1000 mg) may be slightly increased.

8.3 Populations for Analysis

All subjects randomized will be included in the Randomized Set.

The population for all safety analyses is the Safety Analysis Set (SAF). All subjects who receive at least one dose of any investigational product during the study will be included in the SAF population. Subjects will be analyzed according to the treatment received.

Subjects will be included in the Full Analysis Set (FAS) for the primary assessment of activity according to the intent-to-treat principle. The FAS will include all randomized subjects who take at least one dose of investigational product, have at least one activity measurement at baseline and at least one post-baseline study visit with post-baseline activity measurements, and have the presence of keratinocyte K16 expression on baseline biopsies indicative of psoriasis (note that absence of K16 expression at baseline would call into question the underlying diagnosis of psoriasis). Subjects will be analyzed according to the treatment received.

The Per-Protocol Analysis Set (PPS) is defined as all subjects from the FAS set who complete the study and are deemed to be protocol-compliant. This analysis population will only be used if the difference between the FAS population and the PPS population is greater than 10%. To be protocol-compliant, a subject must not have any major protocol deviations during the study period. Protocol deviations will be identified prior to database lock and will be listed by treatment group in the clinical study report. The PPS will be used for a secondary assessment of activity endpoints.

Pharmacokinetics Population is defined as all subjects who receive at least one dose of SRT2104 and have PK data available.

8.4 Procedures for Handling Missing, Unused, and Spurious Data

All available data will be included in data listings and tabulations. Missing values will be imputed using last-observation-carried-forward (LOCF) for the primary analysis of the clinical activity based on biopsy histopathology and for the analyses of PASI-50, PASI-75, and PGA, No other imputation of values for missing data will be performed.

Data that are potentially spurious or erroneous will be examined under the auspices of standard data management operating procedures.

8.5 Statistical Methods

8.5.1 Subject Disposition

The total number of subjects screened, randomized, completed, and prematurely discontinued from the study will be summarized by treatment group and overall. The reason for termination for all subjects who discontinued will be summarized by treatment group and overall. A listing of subjects who discontinued from the study by reason for termination will also be presented.

A table summarizing the analysis sets will be presented, with the number of randomized subjects who satisfy the requirements for inclusion and frequencies of exclusion from the respective SAF, FAS, PPS, and PK analysis sets.

A listing of subjects with major protocol deviations will also be presented.

8.5.2 Subjects Baseline Characteristics

Descriptive summaries of demographic and baseline characteristics will be presented by treatment group for all subjects randomized.

Baseline characteristics will include a summary of the following:

- Subject demographics including age, gender, race;
- Baseline disease characteristics, including duration since diagnosis;
- Pre-existing medical conditions;
- Prior therapies.

8.5.3 Exposure to Investigational Product

Exposure to SRT2104 will be tabulated by group by presenting number of days on study, defined as number of days from day of first dose to day of last dose taken. Total amount of investigational product taken using a similar calculation method will be presented by group.

8.5.4 Safety Analysis

Safety evaluations will be based on the incidence, severity, and type of AEs and clinically significant changes in the subject's physical examination findings, vital signs, and clinical laboratory results. Safety variables will be tabulated and presented for all subjects who receive SRT2104 or placebo (the safety population). Exposure to investigational product and reasons for discontinuation of study treatment will be tabulated.

AEs will be coded using the Medical Dictionary of Regulatory Activities (MedDRA) AE coding system for purposes of summarization. All AEs occurring on study will be listed in by-subject data listings. Treatment-emergent events will be tabulated, where treatment-emergent is defined as any AE that occurs after administration of the first dose of investigational product through 30 days after the last dose of SRT2104, any event that is considered causally drug-related regardless of the start date of the event, or

any event that is present at baseline but worsens in intensity or is subsequently considered drug-related by the investigator. Events that are considered related to treatment (unlikely related, possibly related, or related) will also be tabulated. Tabulation will also be provided that enumerates AEs by maximum severity. Deaths, other SAEs, and events resulting in study discontinuation will be tabulated.

Change from baseline in clinical laboratory parameters will be summarized across time on study. Shift tables may be produced for selected laboratory parameters if implied by the data. Changes in vital sign parameters will be summarized over time in a similar fashion to laboratory parameters, and any abnormal values will be tabulated.

Additional safety analyses may be determined at any time without prejudice, in order to most clearly enumerate rates of toxicities and to further define the safety profile of SRT2104.

8.5.5 Pharmacokinetic and Pharmacokinetic/Pharmacodynamic Analyses

The PK population includes all subjects for which SRT2104 concentration data are available. For the current study, plasma concentrations of SRT2104 for each subject will be listed on the basis of the dose level, time and day at which each sample was collected relative to the first dose. Relevant data from the current study, which may be combined with historical data, will be analyzed using a non-linear mixed effects modeling approach (population PK). Population PK parameters including clearance (CL), volume of distribution (V) and AUC will be estimated. Dependent on the final structural PK model additional PK parameters also may be estimated. Sources of variability in PK parameters will be investigated during population modeling. Demographic or clinical variables including, but not limited to, age, sex, race, and body weight will be evaluated as potential predictors of inter- and intra-subject variability for PK parameters. Results of the population PK model may be used in additional PK/PD analyses. Population modeling will be performed using the non-linear mixed effects modeling software (NONMEM, Globomax LLC; Ellicott City, MD). Further details of population PK analyses will be described under a separate analysis plan to be completed prior to database lock. Results of the population PK analysis will be included in a report separate from the clinical study report.

Measures of individual exposure to SRT2104, such as AUC, may be correlated with selected efficacy endpoints, biomarkers, and measures of safety and tolerability as exploratory analyses of pharmacokinetic/pharmacodynamic relationships. Specific analyses that may be conducted are not specified *a priori*; they will be determined on the basis of study outcome.

8.5.6 Activity Analysis

Activity analyses will be performed to examine the effect of daily doses of SRT2104 on clinical activity in subjects with moderate to severe plaque type psoriasis based on histological assessment of skin biopsies after 12 weeks of exposure. Analyses will also assess the effects of SRT2104 on the PASI in subjects with moderate to severe plaque-type psoriasis after 4, 8, and 12 weeks of exposure. The effects of SRT2104 on the PGA score in subjects with moderate to severe plaque-type psoriasis after 4, 8, and 12 weeks of exposure will also be analyzed.

Exploratory analyses may be performed to characterize expression patterns of select genes and proteins hypothesized to be involved in psoriasis pathophysiology and sirtuin pathways and to evaluate the relationship between these biomarkers and investigational product pharmacokinetics and/or clinical activity. Analyses will also be performed in order to assess the effects of SRT2104 on sense of well-being in subjects with moderate to severe plaque-type psoriasis after 4, 8, and 12 weeks of exposure using the PHQ-9 and the HADS. The potential effects of SRT2104 on health-related quality of life in subjects with moderate to severe plaque-type psoriasis after 12 weeks of exposure using the PQOL-12 will also be analyzed.

Analyses of activity will be performed on the FAS. It may also be performed on the PPS population if the difference between the FAS population and the PPS population is greater than 10%.

Summary statistics for activity variables will be presented for each planned assessment and, for continuous variables, change plus percent change will be presented at each assessment by treatment group. For all subject reported outcomes, appropriate calculations will be made to produce each factor or subscore for each instrument as well as the overall or total instrument score.

For all assessments of activity, the placebo groups will be merged from the 3 cohorts prior to inferential comparisons against active treatments. In addition, the SRT2104 dose cohorts may be merged and serve as an additional group of treated subjects for all activity comparisons.

The primary clinical activity outcome will be on histology (i.e., skin pathology) using the Krueger criteria (see Section 6.4). Point estimates and 90% confidence intervals will be constructed for the differences between the proportion of responders, defined as “good” or “excellent” Krueger improvement score, for each of the exposure groups and the historical null placebo response of 5%.

In secondary efficacy assessments, "response" to treatment will be determined by PASI-50 and PASI-75 response rates. Response is defined as the proportion of subjects who achieve a PASI-50 or PASI-75. Comparisons will also be made for mean change in PASI score, proportion of subjects who achieve "clear" or "almost clear" on the PGA assessment, and proportion of subjects who achieve improvement in PGA by one or more levels. Point estimates and 90% confidence intervals will also be constructed for the secondary endpoints of clinical activity (PASI-50, PASI-75, and PGA of "almost clear" or "clear") for the differences between the proportion of responders for each of the exposure groups and the historical null placebo response of 5%.

The number and percentage of subjects who experience treatment failure as defined in Section 5.2.5 will be displayed by treatment group. Subjects experiencing treatment failure will be identified in the data listings.

The number and percentage of subjects receiving at least one rescue medication (see Section 5.13), will be displayed by treatment group. All rescue medication administrations will be provided in the data listings.

8.6 Procedures for Reporting Deviations to Original Statistical Analysis Plan

A formal statistical analysis plan for the analysis and presentation of data from this study will be prepared before database lock. Deviations from the statistical analyses outlined in this protocol will be indicated in this plan; any further modifications will be noted in the final clinical study report.

9 ADMINISTRATIVE REQUIREMENTS

9.1 Good Clinical Practice

The study will be conducted in accordance with the ICH for GCP and the appropriate regulatory requirement(s). The investigator will be thoroughly familiar with the appropriate use of the investigational product as described in the protocol and IB. Essential clinical documents will be maintained to demonstrate the validity of the study and the integrity of the data collected. The investigator site file and associated study documentation will be archived for up to 30 years. The study documentation may be transferred to an offsite storage facility during this period but will remain under the control of the site. If the sponsor delegates the set-up and maintenance of the sponsor TMF, the TMF will be returned to the sponsor at the end of the study and the sponsor will archive it for up to 30 years after initial marketing approval or termination of development.

9.2 Ethical Considerations

The study will be conducted in accordance with ethical principles founded in the Declaration of Helsinki (see Section 12) and the relevant regulations under 21 CFR parts 312, 50 and 56. The IRB/IEC will review all appropriate study documentation in order to safeguard the rights, safety, and well-being of the subjects. The study will only be conducted at sites where IRB/IEC approval has been obtained. The protocol, Investigator’s Brochure, informed consent, advertisements (if applicable), written information given to the subjects, safety updates, annual progress reports, and any revisions to these documents will be provided to the IRB/IEC by the investigator.

9.3 Subject Information and Informed Consent

After the study has been fully explained, written informed consent will be obtained from either the subject or their guardian or legal representative prior to study participation. The method of obtaining and documenting the informed consent and the contents of the consent will comply with ICH-GCP and all applicable regulatory requirement(s).

9.4 Subject Confidentiality

In order to maintain subject privacy, all CRFs, investigational product accountability records, study reports, and communications will identify the subject by initials and the assigned subject number. The investigator will grant monitor(s) and auditor(s) from Sirtris or its designee and regulatory authority(ies) access to the subject’s original medical records for verification of data gathered on the CRFs and to audit

the data collection process. The subject's confidentiality will be maintained and will not be made publicly available to the extent permitted by the applicable laws and regulations.

9.5 Protocol Compliance

The investigator will conduct the study in compliance with the protocol provided by Sirtris, and given approval/favorable opinion by the IRB/IEC and the appropriate regulatory authority(ies). Modifications to the protocol should not be made without agreement by both the investigator and Sirtris. Changes to the protocol will require written IRB/IEC approval/favorable opinion prior to implementation, except when the modification is needed to eliminate an immediate hazard(s) to subjects. The IRB/IEC may provide, if applicable regulatory authority(ies) permit, expedited review and approval/favorable opinion for minor change(s) in ongoing studies that have the approval /favorable opinion of the IRB/IEC. Sirtris or its designee will submit all protocol modifications to the regulatory authority(ies) in accordance with the governing regulations.

When immediate deviation from the protocol is required to eliminate an immediate hazard(s) to subjects, the investigator will contact Sirtris, if circumstances permit, to discuss the planned course of action. Any departures from the protocol must be fully documented in the CRF and source documentation.

9.6 Study Monitoring

Monitoring and auditing procedures developed by Sirtris will be followed, in order to comply with GCP guidelines. On-site checking of the CRFs for completeness and clarity, cross-checking with source documents, and clarification of administrative matters will be performed.

The study will be monitored by Sirtris or its designee. Monitoring will be done by personal visits from a representative of the sponsor (site monitor) who will review the CRFs and source documents. The site monitor will ensure that the investigation is conducted according to protocol design and regulatory requirements by frequent communications (letter, telephone, and fax).

All unused investigational product and other study materials are to be returned to Sirtris or its designee, or destroyed on site after the clinical phase of the study has been completed (see Section 9.9).

9.7 On-site Audits

Regulatory authorities, the IEC/IRB, and/or Sirtris' clinical quality assurance group may request access to all source documents, CRFs, and other study documentation for on-site audit or inspection. Direct access

to these documents must be guaranteed by the investigator, who must provide support at all times for these activities.

9.8 Case Report Form Completion

Case report forms will be completed for each study subject. It is the investigator's responsibility to ensure the accuracy, completeness, legibility, and timeliness of the data reported in the subject's CRF. Source documentation supporting the CRF data should indicate the subject's participation in the study and should document the dates and details of study procedures, AEs, and subject status.

The investigator, or designated representative, should complete the CRF pages as soon as possible after information is collected, preferably on the same day that a study subject is seen for an examination, treatment, or any other study procedure. Any outstanding entries must be completed immediately after the final examination. An explanation should be given for all missing data.

9.9 Drug Accountability

Accountability for the investigational product at the study site and with the subject is the responsibility of the investigator. The investigator will ensure that the investigational product is used only in accordance with this protocol. Where allowed, the investigator may choose to assign some of the drug accountability responsibilities to a pharmacist or other appropriate individual. Drug accountability records indicating the drug's delivery date to the site, inventory at the site, use by each subject, and return to Sirtris (or destruction and disposal of the drug, if approved by Sirtris) will be maintained by the clinical site. These records will adequately document that the subjects were provided the doses as specified in the protocol and should reconcile all investigational product received from Sirtris. Accountability records will include dates, quantities, batch/serial numbers, expiration dates (if applicable), and subject numbers. The sponsor or its designee will assign a site monitor to review drug accountability at the site on an ongoing basis during monitoring visits.

All unused and used investigational product will be retained at the site until they are inventoried by the site monitor. All used, unused or expired investigational product will be returned to Sirtris or its designee, or if authorized, disposed of at the study site and documented. All material containing SRT2104 will be treated and disposed of as hazardous waste in accordance with governing regulations.

9.10 Premature Closure of the Study

This study may be prematurely terminated, if in the opinion of the investigator or Sirtris, there is sufficient reasonable cause. Written notification documenting the reason for study termination will be provided to the investigator or Sirtris by the terminating party.

Circumstances that may warrant termination include, but are not limited to:

- Determination of unexpected, significant, or unacceptable risk to subjects;
- Failure to enter subjects at an acceptable rate;
- Insufficient adherence to protocol requirements;
- Insufficient complete and/or evaluable data;
- Plans to modify, suspend or discontinue the development of the investigational product.

Should the study be closed prematurely, all study materials must be returned to Sirtris.

9.11 Record Retention

The investigator will maintain all study records according to ICH-GCP, applicable regulatory requirement(s) and Sirtris' record retention policy. Records will be retained for 30 years after the last marketing application approval or 30 years after formal discontinuation of the clinical development of the investigational product or according to applicable regulatory requirement(s). The study documentation may be transferred to an offsite storage facility during this period but will remain under the control of the site. If the investigator withdraws from the responsibility of keeping the study records, custody must be transferred to a person willing to accept the responsibility. Sirtris must be notified in writing in advance of any change in disposition of the study records, including if a custodial change occurs.

9.12 Sample Disposition

Samples collected for safety analyses will be destroyed by the central laboratory approximately 15 days after the analysis is completed. The PK samples will be stored at Simbec Research in Merthyr Tydfil in the United Kingdom for up to 2 years after the last subject completes the study. The skin biopsy tissue samples will be stored at the Rockefeller University in New York, NY for up to 2 years after the last subject completes the study. At the end of the 2-year interval, the samples will, at the directive of Sirtris

Pharmaceuticals either be destroyed, or will be transferred to GSK where they will be stored for up to 15 years after the last subject completes the study.

9.13 Liability and Insurance

Sirtris will be subscribed to an insurance policy covering, in its terms and provisions, its legal liability for injuries caused to participating persons and arising out of this research performed strictly in accordance with the scientific protocol as well as with applicable law and professional standards.

10 USE OF INFORMATION

All information regarding SRT2104 supplied by Sirtris to the investigator is privileged and confidential information. The investigator agrees to use this information to accomplish the study and will not use it for other purposes without consent from Sirtris. It is understood that there is an obligation to provide Sirtris with complete data obtained during the study. The information obtained from the clinical study will be used towards the development of SRT2104 and may be disclosed to regulatory authority(ies), other investigators, corporate partners, or consultants as required.

Publication of the results of the study, whether in whole or in part, shall be within the sole and absolute discretion of Sirtris. Investigators, sites, CROs and/or designees shall not be entitled to publish any of the data or information arising during or out of the provision of the services without the prior written consent of Sirtris. For the avoidance of doubt Sirtris reserves the unqualified right to reject any paper or article utilizing any data generated from this study before such paper or article is presented or submitted for publication.

11 INVESTIGATOR AGREEMENT

I have read the Protocol entitled, "A Randomized, Double-Blind, Placebo-Controlled, Phase IIa Study of the Clinical Activity, Safety, and Tolerability of SRT2104 in Subjects with Moderate to Severe Plaque-Type Psoriasis"

I agree to conduct the study as detailed herein and in compliance with ICH Guidelines for Good Clinical Practice and applicable regulatory requirements and to inform all who assist me in the conduct of this study of their responsibilities and obligations.

____

Principal Investigator (Printed Name)

____ ____________________

Principal Investigator Signature Date

____

____

____

Investigational site or name of institution and location (printed)

12 REFERENCES

Blander G, Guarente L. The Sir2 family of protein deacetylases. *Annu Rev Biochem.* 2004; 73:417-435.

Bouras T, Fu M, Sauve AA, et al. SIRT1 deacetylation and repression of p300 involves lysine residues 1020/1024 within the cell cycle regulatory domain 1. *J Biol Chem.* 2005 Mar; 280:10264-76.

Brunet A, et al. Stress-dependent regulation of FOXO transcription factors by the SIRT1 deacetylase. *Science.* 2004; 303:2011-2015.

Brakenhielm, E, et al. Suppression of angiogenesis, tumor growth, and wound healing by resveratrol, a natural compound in red wine and grapes. *Faseb J.* 2001 Aug, 15:1798-1800.

Cohen H, Miller C, Bitterman K, et al. Calorie restriction promotes mammalian cell survival by inducing the SIRT1 deacetylase. *Science.* 2004 Jul; 305(5682):390-392.

Corton JC, Brown-Borg HM. Peroxisome proliferator-activated receptor gamma coactivator 1 in caloric restriction and other models of longevity. *J Gerontol A Biol Sci Med Sci.* 2005 Dec; 60:1494-1509.

Feldman SR, et al. Decision points for the initiation of systemic treatment for psoriasis. *Journal of the American Academy of Dermatology*. 2005; 53:101

Fontana L. Neuroendocrine factors in the regulation of inflammation: excessive adiposity and calorie restriction. *Experimental Gastroenterology*. 2009; 44:41-45.

Fortune DG, et al. Quality of life in patients with psoriasis: the contribution of clinical variables and psoriasis. *British Journal of Dermatology*. 1997; 137: 755-760

Frye RA. Characterization of five human cDNAs with homology to the yeast SIR2 gene: Sir2-like proteins (sirtuins) metabolize NAD and may have protein ADP-ribosyltransferase activity. *Biochem. Biophys. Res. Commun.* 1999; 260:273-279.

Frye RA. Phylogenetic classification of prokaryotic and eukaryotic Sir2-like proteins. *Biochem. Biophys. Res. Commun.* 2000; 273:793-798.

Handschin C, Spiegelman, BM. The role of exercise and PGC1-α in inflammation and chronic disease. *Nature.* 2008; 454(7203):463-469

Heilbronn LK, Ravussin E. Calorie restriction and aging: review of the literature and implications for studies in humans. *Am J Clin Nutr.* 2003 Sep; 78:361-369.

Heilbronn LK, Ravussin E. Calorie restriction extends life span-but which calories? *PLoS Med.* 2005 Aug; 2:e231.

Holian, O, Walter RJ. Resveratrol inhibits the proliferation of normal human keatinocytes in vitro. *J Cell Biol.* 2001; Supplement 36:55-62

Imai S, Armstrong CM, Kaeberlein M. Transcriptional silencing and longevity protein Sir2 is an NAD-dependent histone deacetylase. *Nature.* 2000 Feb; 403:795-800.

Ingram DK, Zhu M, Mamczarz J, et al. Calorie restriction mimetics: an emerging research field. *Aging Cell.* 2006; 5:97-108.

Koo J, Population-based epidemiologic study of psoriasis with emphasis on quality of life assessment. *Dermatol Clin.* 1996; 485-96)

Koo J, et al. The Development of a Disease-Specific Questionnaire to Assess Quality of Life for Psoriasis Patients: An Analysis of the Reliability, Validity, and Responsiveness of the Psoriasis Quality of Life Questionnaire. *Dermatol. Psychosom*. 2002; 3:171–179.

Kroenke K, Spitzer RL, Williams JBW. The PHQ-9: Validity of a Brief Depression Severity Measure. *J Gen Intern Med*; 2001 16(9): 606–613.

Krueger JG, et al. A human interleukin-12/23 monoclonal antibody for the treatment of psoriasis. *NEJM.* 2007; 356:580

Luo J, Nikolaev AY, Imai S, et al. Negative control of p53 by Sir2alpha promotes cell survival under stress. *Cell.* 2001 Oct; 107:137-148.

Mykletun A, et.al.. Hospital Anxiety and Depression (HAD) scale: factor structure, item analyses and internal consistency in a large population. *British Journal of Psychiatry*, 2001; 179: 540

Milne J, Lambert P, Schenk S, et al. Small molecule activators of SIRT1 as therapeutics for the treatment of type 2 diabetes. *Nature.* 2007 Nov; 450:712-716.

Motta MC, Divecha N, Lemieux M, et al. Mammalian SIRT1 represses forkhead transcription factors. *Cell.* 2004 Feb; 116:551-563.

Nemoto S, Fergusson MM, Finkel T. SIRT1 functionally interacts with the metabolic regulator and transcriptional coactivator PGC-1α. *J Biol Chem.* 2005 Apr; 280:16456-16460.

Nisoli E, Tonello C, Cardile A, et al. Calorie restriction promotes mitochondrial biogenesis by inducing the expression of eNOS. *Science.* 2005 Oct; 310:314-317.

Picard F, Kurtev M, Chung N, et al. Sirt1 promotes fat mobilization in white adipocytes by repressing PPAR-gamma. *Nature.* 2004 Jun; 429:771-776.

Rapp SR, et al. Psoriasis causes as much disability as other major medical diseases. *J Am Acad Dermatol.* 1999; 41:401-7

Rodgers J, Lerin C, Haas W, et al. Nutrient control of glucose homeostasis through complex of PGC-1α and SIRT1. *Nature.* 2005 Mar; 434(7029):113-118.

Roth, G. S., Ingram, D. K. & Lane, M. A. Caloric restriction in primates and relevance to humans. *Ann N Y Acad Sci.* 2001; 928:305-315.

Schon MP, Boehncke WH. Psoriasis. *NEJM.* 2005; 352:1899

Smith J, et al. Small molecule activators of SIRT1 replicate signaling pathways triggered by calorie restriction in vivo. *BMC Systems Biology*. 2009; 3:31.

van der Horst A, et al. FOXO4 is acetylated upon peroxide stress and deacetylated by the longevity protein hSir2(SIRT1). *J Biol Chem.* 2004; 279, 28873-28879.

Vaziri H, Dessain SK, Ng Eaton E, et al. hSIR2(SIRT1) functions as an NAD-dependent p53 deacetylase. *Cell.* 2001 Oct; 107:149-159.

Weindruch R, Keenan KP, Carney JM et al. Caloric restriction mimetics: metabolic interventions. *J Gerontol A Biol Sci Med Sci.* 2001 Mar; 56 Spec No 1:20-33.

World Medical Association Declaration of Helsinki : Ehtical Principles for Medical Research Involving Human Subjects. October, 2008. http://www.wma.net/en/30publications/10policies/b3/index.html

Yeung F, Hoberg JE, Ramsey CS, et al. Modulation of NF-kappaB-dependent transcription and cell survival by the SIRT1 deacetylase. *EMBO J.* 2004 Jun; 23:2369-80.

Yoshizaki T, et al. SIRT1 Inhibits Inflammatory Pathways in Macrophages and Modulates Insulin Sensitivity. *Am J Physiol Endocrinol Metab*. Dec, 2009.

13 Appendix 1 List of Permitted Rescue Medications

Class 6 steroids
Alclometasone dipropionate 0.05% cream and 0.05% ointment
Desonide 0.05% cream, 0.05% foam, 0.05% gel and 0.05% lotion
Fluocinolone acetonide 0.01% shampoo and 0.01% solution
Flurandrenolide 0.025% cream
Triamcinolone acetonide 0.025% cream and 0.025% lotion

Class 7 steroids
Hydrocortisone 0.5% cream and 0.5% ointment
Hydrocortisone 1% cream, 1% lotion and 1% ointment
Hydrocortisone 2.5% cream, 2.5% lotion and 2.5% ointment

14 Appendix 2 Pharmacogenetic Research

Background

Pharmacogenetics (PGx) is the study of variability in drug response due to hereditary factors in different populations. There is increasing evidence that an individual's genetic composition (i.e., genotype) may impact the pharmacokinetics (absorption, distribution, metabolism, and elimination), pharmacodynamics (relationship between concentrations and pharmacologic effects or the time course of pharmacologic effects) and/or clinical outcome (in terms of efficacy and/or safety and tolerability). Some reported examples of PGx analysis include:

Drug	Disease	Gene	Outcome
Abacavir	HIV [Hetherington, 2002; Mallal, 2002]	HLA (human leukocyte antigen)	Caucasian males with HLA B57 variant were at increased risk for experiencing hypersensitivity to abacavir
Tranilast	Restenosis prevention following coronary bypass [Roses, 2002]	UGT1A1	Drug induced hyperbilirubinemia explained by high proportion of affected patients having 7/7 TA repeat genotype, consistent with clinically benign Gilbert’s Syndrome
ABT-761	Asthma [Drazen, 1999]	ALOX5	ALOX5 Sp1 promoter genotype (x,x) associated with reduced response to 5-lipoxygenase inhibitor ABT-761

A key component to successful PGx research is the collection of samples during the conduct of clinical studies.

Collection of whole blood samples, even when no a priory hypothesis has been identified, may enable PGx analysis to be conducted if at any time it appears that there is a potential unexpected or unexplained variation in handling or response to SRT2104.

Pharmacogenetics Research Objectives

The objective of the PGx research (if there is a potential unexpected or unexplained variation) is to investigate a possible genetic relationship to handling or response to SRT2104. If at any time it appears there is potential variability in response in this clinical study or in a series of clinical studies with SRT2104 that may be attributable to genetic variations of subjects, the following objectives may be investigated:

- Relationship between genetic variants and the pharmacokinetics of investigational product

- Relationship between genetic variants and safety and/or tolerability of investigational product
- Relationship between genetic variants and efficacy of investigational product.

Study Population

Any subject who has given informed consent to participate in the clinical study, has met all the entry criteria for the clinical study, and receives investigational product may take part in the PGx research. Any subject who has received an allogeneic bone marrow transplant must be excluded from the PGx research.

Subject participation in the PGx research is voluntary and refusal to participate will not indicate withdrawal from the clinical study. Refusal to participate will involve no penalty or loss of benefits to which the subject would otherwise be entitled.

Study Assessments and Procedures

In addition to any blood samples taken for the clinical study, a whole blood sample (~10ml) will be collected for the PGx research using a tube containing EDTA. The PGx sample is labelled (or coded) with a study specific number that can be traced or linked back to the subject by the investigator or site staff. Coded samples do not carry personal identifiers (such as name or social security number). The blood sample will be taken on a single occasion unless a duplicate sample is required due to inability to utilize the original sample. It is recommended that the blood sample be taken at the first opportunity after a subject has been randomized and provided informed consent for PGx research, but may be taken at any time while the subject is participating in the clinical study.

If deoxyribonucleic acid (DNA) is extracted from the blood sample, the DNA may be subjected to sample quality control analysis. This analysis will involve the genotyping of several genetic markers to confirm the integrity of individual samples. If inconsistencies are noted in the analysis, then those samples may be destroyed.

The need to conduct PGx analysis may be identified after a study (or set of studies) of SRT2104 has been completed and the study data reviewed.

In some cases, the samples may not be studied e.g., no questions are raised about how people respond to SRT2104.

Samples will be stored securely and may be kept for up to 30 years after the last subject completes the study or Sirtris may destroy the samples sooner. Sirtris or those working with Sirtris (for example, other

researchers) will use samples collected from the study for the purpose stated in this protocol and in the informed consent form.

Subjects can request their sample to be destroyed at any time.

Provision of Study Results and Confidentiality of Subject's PGx Data

Sirtris may summarize the cumulative PGx research results in the clinical study report. In general, Sirtris does not inform the investigator, subject or anyone else (e.g., family members, study investigators, primary care physicians, insurers, or employers) of the PGx research results because the information generated from PGx studies is preliminary in nature, and the significance and scientific validity of the results are undetermined at such an early stage of research, under any circumstance unless required by law.

References

Drazen JM, Yandava CN, Dube L, Szcerback N, Hippensteel R, Pillari A, Israel E, Schork N, Silverman ES, Katz DA, Drajesk J. Pharmacogenetic association between ALOX5 promoter genotype and the response to anti-asthma treatment. *Nature Genet*. 1999; 22:168-70.

Hetherington S, Hughes AR, Mosteller M, Shortino D, Baker KL, Spreen W, Lai E, Davies K, Handley A, Dow DJ, Fling ME, Stocum M, Bowman C, Thurmond LM, Roses AD. Genetic variations in HLA-B region and hypersensitivity reactions to abacavir. *Lancet*. 2002; 359:1121-2.

Mallal S, Nolan D, Witt C, Masel G, Martin AM, Moore C, Sayer D, Castley A, Mamotte C, Maxwell D, James I. Association between presence of HLA-B*5701, HLA-DR7, and HLA-DQ3 and hypersensitivity to HIV-1 reverse-transcriptase inhibitor abacavir. *Lancet*. 2002; 359:727-32.

Roses AD. Genome-based pharmacogenetics and the pharmaceutical industry. *Nat Rev Drug Discov*. 2002; 1:541-9.

15 Appendix 3 Summary of Changes to Amendment 1

**Detailed description of changes**	
**Section**	CHANGES (new text indicated in **bold**, removed text indicated as ~~strikethrough~~):
**Section 1.1, Schedule of Events**	Tuberculosis (TB) test was added to Screening Visit. Original version of the protocol included an exclusion criterion of a positive TB test at the Screening visit, however the TB test was inadvertently omitted from the Schedule of Events in the original protocol.
**Section 5.15 Contraceptive Use**	First sentence modified:<br><br>All female subjects of child-bearing potential must use ~~two~~ **an** adequate form~~s~~ of contraception for the duration of the trial (from the Screening Visit through Day 114).
**Section 5.2.1, Number of Subjects**	The text was modified:<br><br>A sample size of ~~10~~ **8** evaluable subjects per active treatment group and 6 evaluable subjects in the merged placebo group is based on **feasibility.** ~~the following assumptions: an exact binomial test with a nominal 0.05 one-sided significance level will have 80% power to detect the difference between a null placebo success rate of 10.0% and a SRT2104 histology success rate (response of excellent) of 33.0%.~~ Subjects who withdraw from the study prior to 8 weeks of treatment may or may not be replaced (see Section 5.2.2).
**Section 5.2.3, Inclusion Criteria**	The first sentence was modified:<br>A subject will be eligible for inclusion in this study only if all of the following Screening **and baseline PASI** criteria are met:<br><br>Inclusion Criterion # 6 was modified:<br>If a female subject of child-bearing potential, be willing to use reliable contraception (see Section 5.15) for the duration of the study, through the 30 day safety follow up ~~telephone call~~ **visit (a female of child-bearing potential is defined as any female, regardless of her age with functioning ovaries and no documented impairment of oviductal or uterine function that would cause sterility. Females with oligomenorrhea or who are perimenopausal, and young females who have begun to menstruate are considered to be of child-bearing potential)**
**Section 5.11, Safety Review and Dose Escalation or Cessation**	The second and third paragraph were modified:<br>All safety data generated for all subjects **in the 250 mg and 500 mg** ~~every~~ cohort**s** who have completed at least 28 days of dosing will be reviewed by the ISRC. In the event of clinically significant adverse

Detailed description of changes	
**Section**	CHANGES (new text indicated in **bold**, removed text indicated as ~~strikethrough~~):
	events deemed to be of sufficient severity to pause dosing **(refer to criteria in Section 5.11.1)**, a full analysis of all safety data will be conducted before continuing with a given dose or with dose escalation.<br>When the last subject in **the applicable** ~~a~~ cohort has completed 28 days of dosing, the data for each subject will be manually entered into an electronic data capture system. The laboratory data will be transferred electronically into the database. The safety data will then be listed and presented to the ISRC for review. If the safety data is acceptable, any subjects still active in the current cohort will continue dosing, and the ISRC will authorize the initiation of dosing to the next cohort of subjects at the higher dose level. This will be repeated until **the first 2** ~~all three dosing~~ cohorts have completed at least 28 days of dosing.

Detailed description of changes	
**Section**	CHANGES (new text indicated in **bold**, removed text indicated as ~~strikethrough~~):
**Section 5.11.1, Criteria for discontinuing dosing/dose escalation**	Section modified:<br><br>In the event that there are **SAEs or severe AEs reported** ~~any serious adverse events or moderate to severe adverse event toxicities~~ reported in a cohort **in which a possible relationship to investigational product cannot be fully excluded** , one of the following procedures will be applied:<br>• If one subject receiving **SRT2104** ~~test material~~ in a cohort **experiences a severe AE or an SAE,** ~~is intolerant of the test material,~~ but the other subjects receiving that same dose are tolerant of that dose, the ISRC and Sponsor will determine whether the nature, severity, or the number of **AEs** ~~toxicities~~ permit continuing the dosing cohort, or if dosing and/or dose escalation will stop.<br>• If at least two subjects **receiving** ~~are intolerant of a given dose of~~ SRT2104 **in a cohort experience a severe AE or an SAE**, but similar **AEs** ~~toxicities~~ are recorded for subjects on placebo, the ISRC and Sponsor will determine whether the nature, severity, or the number of **AEs** ~~toxicities~~ permit continuing the dosing cohort, or if dosing and/or dose escalation will stop.<br>If at least two subjects **receiving SRT2104 in a cohort experience a severe AE or an SAE,** ~~are intolerant of SRT2104 at a given dose level~~, and no **severe AEs or SAEs** ~~toxicities~~ are seen in placebo subjects, the **dosing of new subjects and/or** dose escalation will **be halted pending full review by the GSK Global Safety Board.** ~~stop.~~
**Section 5.14, Permitted Medications**	A fourth bullet has been added:<br><br>**If the investigator desires to initiate therapy with a prescription medication (e.g., in the event of a newly diagnosed medical condition) during the dosing period, s/he should contact the Medical Monitor to discuss the new therapy prior to initiating it.**
**Section 6, Study Procedures**	The first paragraph has been modified:<br><br>The timepoints for all study procedures are reflected in the Schedule of Events (see Section 1.1). ~~All visits and telephone contacts will be scheduled to take place as near as possible to the scheduled day.~~ **Visit windows are as follows: a plus/minus 4-day window will be applied to Visits 3 through 7; a 1-day plus/minus window will be applied to Visit 8 (Day 84).**

**Detailed description of changes**	
**Section**	CHANGES (new text indicated in **bold**, removed text indicated as ~~strikethrough~~):
	New section added:<br><br>Tuberculosis Test<br>A tuberculosis test will be performed at the Screening Visit. A positive test result that cannot be attributed to a prior BCG inoculation will disqualify the subject. (see Section 5.2.4)
**Section 6.2, Pharmaco-kinetic Sampling**	The 4th sentence has been modified:<br><br>A total of five blood samples (~~4~~ **6** mL each) will be obtained from each subject over the course of the study for determination of SRT2104 plasma concentrations.
**Section 7.1.5, Liver Chemistry Stopping Rules and Follow Up**<br>**Section 7.2 Procedures for Recording and Reporting AEs and SAEs**	References to reporting to Akos Limited have been deleted.<br><br>SAE/SUSAR Reporting Contact Information modified:<br><br>~~Akos Limited~~<br>~~The Coach House, Pipers Lane~~<br>~~Harpenden, Hertfordshire~~<br>~~AL5 1AH United Kingdom~~<br>~~SAE Reporting Line: 011 44 1582 761888 or~~<br>~~PVSirtris@akos.co.uk~~<br>~~Telephone: 011 44 1582 766339~~<br><br>Sirtris Pharmacovigilance<br>Sirtris Pharmaceuticals<br>200 Technology Square<br>Cambridge, MA 02139 USA<br>**Fax Line: 1 617 679 8499 Or**<br>**Email: SirtrisPVG@sirtrispharma.com**
**Section 7.4, Pregnancy Reporting**	Text following 3rd sentence modified:<br><br>The outcome of the pregnancy will also be followed. **Any premature termination of the pregnancy will be reported.** Information on the status of the mother and child will be forwarded to Sirtris **at the time of birth, where applicable**. Generally, follow-up **of live births** will occur at approximately 3-month intervals and

Detailed description of changes	
**Section**	CHANGES (new text indicated in **bold**, removed text indicated as ~~strikethrough~~):
	will be no longer than 12 months following the estimated delivery date. ~~Any premature termination of the pregnancy will be reported.~~
**Section 8, Statistical Procedures**	Text modified:<br><br>~~The sample size has been chosen based on the probability of overall response to treatment and feasibility to allow preliminary characterization of safety, tolerability, and PK and to explore pharmacodynamic measures.~~<br>~~Alphas will be two-tailed in nature and set at 0.05 for all analyses unless otherwise stated. As this is a proof of concept study, no adjustment for multiple analyses will be made.~~<br><br>**The primary objectives are (1) to assess the effects of SRT2104 on clinical activity, based on the Krueger criteria, relative to a historical placebo response rate of 5% and (2) to assess safety and tolerability of SRT 2104. For the first objective the analysis will be based on an exposure-response analysis. Previous studies have indicated that the pharmacokinetic exposure is relatively variable, for this reason, the active treatments will be combined and then dichotomized into a low and high drug exposure group. The cut-off point will likely be the midpoint of exposure but, the selection of the final cut-point will be dependent on the distribution of exposure. Point estimates and 90% confidence intervals will be constructed for the differences between the proportion of responders, defined as "good" or "excellent" Krueger improvement score, for each of the exposure groups and the historical null placebo response of 5%. No formal hypothesis testing will be performed. The second primary objective will be accomplished by review of individual subject data and descriptive summaries of the safety data. Safety displays will be summarized by treatment group (placebo, 250mg, 500mg, and 1000mg). Point estimates and 90% confidence intervals will also be constructed for the secondary endpoints of clinical activity (PASI-50, PASI-75, and PGA of "almost clear" or "clear") for the differences between the proportion of responders for each of the exposure groups and the historical null placebo response of 5%. The clinical activity endpoints will also be summarized by treatment group, as a secondary analysis. All other pharmacodynamic endpoints will summarized by treatment group. Point estimates and corresponding 90% confidence intervals will be constructed for PK comparisons.**

Detailed description of changes

Section	CHANGES (new text indicated in **bold**, removed text indicated as ~~strikethrough~~):
**Section 8.2, Sample Size**	Text modified as follows:

A sample size of 8 evaluable subjects per active treatment group and 6 evaluable **placebo** subjects in **a** ~~the~~ merged placebo group is based on **feasibility. T**he following **information is provided to estimate the precision of the estimates, assuming** ~~assumptions: an exact binomial test with a nominal 0.05 one-sided significance level will have 80% power to detect the difference between a null placebo success rate of 10.0% and a~~ SRT2104 histology success rate (an improvement score of "**good or** excellent**"** ~~improvement"~~ based on the Krueger criteria [see Section 6.4]) of 33.0% **and the exposure data is dichotomized at the midpoint (n=12 for each exposure group).**

Time Krueger response rate	Null placebo response rate	Power	Cut-off value for rejecting Ho	Difference between true and null response rate	90% CI for the difference*
.33	.05	.8124	3	.28	(0.1229,0.6084)
				.20	
.33	.10	.5973	4	.23	(0.1230,0.6090)
				.15	

***90% CI is based on exact methods**

Note, hypothesis testing was performed, instead of an estimation approach, based on the twelve subjects: a single sample exact binomial test with a nominal 0.05 one-sided significance level will have 82% power to detect the difference from the between a null placebo response success rate of 5%, given the and a true SRT2104 histology success response rate (an improvement score of "excellent improvement" based on the Krueger criteria [see Section 6.4]) of 33.0%. Subjects who withdraw from the study prior to 8 weeks of treatment may or may not be replaced (see Section 5.2.2)

In addition, with a sample size of 8 for active dose groups, the probability of observing at least one adverse event of a given type will be 72.8% when the probability of such an adverse event is actually 15.0%.

Based on the results of the second cohort (SRT2104 500 mg) the sample size of the last cohort (SRT2104 1000 mg) may be slightly increased.

Detailed description of changes	
**Section**	CHANGES (new text indicated in **bold**, removed text indicated as ~~strikethrough~~):
**Section 8.3, Populations for Analysis**	The 3rd and 4th paragraphs were modified:<br><br>Subjects will be included in the Full Analysis Set (FAS) for the primary assessment of activity according to the intent-to-treat principle. The FAS will include all randomized subjects who take at least one dose of investigational product ~~and~~ have at least one activity measurement at baseline and at least one post-**baseline** ~~randomization~~ study visit **with post-baseline activity measurements and have the presence of keratinocyte K16 expression on baseline biopsies indicative of psoriasis (note that absence of K16 expression at baseline would call into question the underlying diagnosis of psoriasis).** . Subjects will be **analyzed according to the treatment received**~~. included in the treatment group to which they were randomized.~~<br><br>The Per-Protocol Analysis Set (PPS) is defined as all subjects from the FAS set who complete the study and are deemed to be protocol-compliant. **This analysis population will only be used if the difference between the FAS population and the PPS population is greater than 10%.**
**Section 8.4, Procedures for Handling Missing, Unused, and Spurious Data**	The 2nd and 3rd paragraphs were modified:<br><br>All available data will be included in data listings and tabulations. **Missing values will be imputed using last-observation-carried-forward (LOCF). The primary analysis of the clinical activity based on biopsy histopathology and for the analyses of PASI-50, PASI-75, and PGA.** No **other** imputation of values for missing data will be performed.<br><br>~~Percentages of subjects with AEs or laboratory toxicities will be based on non-missing values.~~
**Section 8.5.6, Activity Analysis**	Modifications were made beginning with the 3rd paragraph:<br><br>Analyses of activity will be performed on ~~both~~ the FAS. **It may also be performed on the** ~~and~~ PPS population~~s~~ **if the difference between the FAS population and the PPS population is greater than 10%.**<br><br>Summary statistics for activity variables will be presented for each planned assessment and, for continuous variables, change plus percent change will be presented at each assessment by treatment

Detailed description of changes	
**Section**	CHANGES (new text indicated in **bold**, removed text indicated as ~~strikethrough~~):
	group. For all subject reported outcomes, appropriate calculations will be made to produce each factor or subscore for each instrument as well as the overall or total instrument score.<br><br>For all assessments of activity, the placebo groups will be merged from the 3 cohorts prior to inferential comparisons against active treatments. In addition, the ~~two highest~~ SRT2104 dose cohorts may be merged and serve as an additional group of treated subjects for all activity comparisons.<br><br>The primary clinical activity outcome will be on histology (i.e., skin pathology) using the Krueger criteria (see Section 6.4). **Point estimates and 90% confidence intervals will be constructed for the differences between the proportion of responders, defined as "good" or "excellent" Krueger improvement score, for each of the exposure groups and the historical null placebo response of 5%.** ~~The proportion of subjects with an improvement score of "excellent improvement" will be compared against the null placebo response of 10% using single-sided, one sample binomial tests.~~<br><br>In secondary efficacy assessments, "response" to treatment will be determined by PASI-50 and PASI-75 response rates. Response is defined as the proportion of subjects who achieve a PASI-50 or PASI-75. Comparisons will also be made for mean change in PASI score, proportion of subjects who achieve "clear" or "almost clear" on the PGA assessment, and proportion of subjects who achieve improvement in PGA by one or more levels. **Point estimates and 90% confidence intervals will also be constructed for the secondary endpoints of clinical activity (PASI-50, PASI-75, and PGA of "almost clear" or "clear") for the differences between the proportion of responders for each of the exposure groups and the historical null placebo response of 5%.** ~~Response rates for the active treatment groups will be compared against the null placebo response of 10% using single sided, one sample binomial tests~~.<br><br>~~For all other activity assessments, analysis of baseline to each assessment differences will be performed employing analyses of covariance (ANCOVA) on values, changes, and percent changes. Baseline values will be used as the covariate for these analyses. If distributions are found to deviate significantly from normal, non-parametric analyses will be substituted for ANCOVA. For variables where change from baseline and percent change from baseline are not appropriate, t-tests will be performed to assess difference~~

Detailed description of changes	
**Section**	CHANGES (new text indicated in **bold**, removed text indicated as ~~strikethrough~~):
	~~between groups.~~<br><br>The number and percentage of subjects who experience treatment failure as defined in Section 5.2.5 will be displayed by treatment group. ~~The proportion of subjects who experience treatment failure will be inferentially assessed by employing Fisher's Exact tests for potential differences between groups.~~ Subjects experiencing treatment failure will be identified in the data listings.<br><br>The number and percentage of subjects receiving at least one rescue medication (see Section 5.13), will be displayed by treatment group. ~~The proportion of subjects who experience treatment failure will be inferentially assessed by employing Fisher's Exact tests for potential differences between groups~~. All rescue medication administrations will be provided in the data listings.
**Section 9.12, Sample Disposition**	New section added. Subsequent sections are re-numbered.<br><br>**Samples collected for safety analyses will be destroyed by the central laboratory approximately 15 days after the analysis is completed. The PK samples will be stored at Simbec Research in Merthyr Tydfil in the United Kingdom for up to 2 years after the last subject completes the study. The skin biopsy tissue samples will be stored at the Rockefeller University in New York, NY for up to 2 years after the last subject completes the study. At the end of the 2-year interval, the samples will, at the directive of Sirtris Pharmaceuticals either be destroyed, or will be transferred to GSK where they will be stored for up to 15 years after the last subject completes the study.**
