# Supplementary material for: A Randomized, Placebo-Controlled Study of SRT2104, a SIRT1 Activator, in Patients with Moderate to Severe Psoriasis
Source: PLoS One. 2015 Nov 10;10(11):e0142081. doi: 10.1371/journal.pone.0142081 (PMC4640558; doi:10.1371/journal.pone.0142081)
Supplement: S1 Table — (DOCX) [file pone.0142081.s004.docx]

SUPPLEMENTAL DATA:

**S1 Table**. **SRT2104 Pharmacokinetic Parameters by Treatment and Exposure groups**

| Treatment (n) | AUC | | Cmax | |
| --- | --- | --- | --- | --- |
|  | Mean  (SD) | Median  (Min, Max) | Mean  (SD) | Median  (Min, Max) |
| SRT2104 250 mg (9) | 2148 (1247) | 2114 (144.3, 4497) | 262(134) | 285 (6.0, 434) |
| SRT2104 500 mg (11) | 4189 (2143) | 3892 (1387, 8870) | 407 (238) | 452 (64.2, 720) |
| SRT2104 1000 mg (11) | 8358 (7467) | 6097 (367, 27355) | 1006 (813) | 626 (23.5, 2442) |
|  |  |  |  |  |
| Low exposure^1^ (15) | 2064 (1052) | 2072 (144.3, 3563) | 224 (144) | 192 (6.0, 447) |
| High exposure^1^ (16) | 7900 (6006) | 5856 (3892, 27355) | 908 (665) | 629 (401, 2442) |

^1^ Exposure categories were based on AUC
