# Supplementary material for: A Randomized, Placebo-Controlled Study of SRT2104, a SIRT1 Activator, in Patients with Moderate to Severe Psoriasis
Source: PLoS One. 2015 Nov 10;10(11):e0142081. doi: 10.1371/journal.pone.0142081 (PMC4640558; doi:10.1371/journal.pone.0142081)
Supplement: S2 Table — (DOCX) [file pone.0142081.s005.docx]

SUPPLEMENTAL DATA:

S2 Table. Proportion of Subjects Improvement Based on PGA Score – Day 84

| Treatment Arm | Proportion of Subjects with PGA Score of Clear or Minimal | | 90% CI for Difference of Placebo vs. Active Treatment (%) |
| --- | --- | --- | --- |
|  | n | # (%) |  |
| Placebo^1^  N=7 | 5 | 0 | na |
| 500 mg SRT2104^1^  N=12 | 9 | 2 (22.2)0 | (16.67, 28.89) |
| 1000 mg SRT2104  N=11 | 11 | 3 (27.3) | (22.73, 32.49) |
| All Active  N=32 | 20 | 5 (25.0) | (22.50, 30.08) |
| Low Exposure  N=15 | 7 | 0 | (-10.00, 6.01) |
| High Exposure  N=16 | 13 | 5 (38.5%) | (34.62, 42.42) |

^1^ No PGA scores were available for patients in SRT2104 250 mg cohort due to an operational error
