# Supplementary material for: A Randomized, Placebo-Controlled Study of SRT2104, a SIRT1 Activator, in Patients with Moderate to Severe Psoriasis
Source: PLoS One. 2015 Nov 10;10(11):e0142081. doi: 10.1371/journal.pone.0142081 (PMC4640558; doi:10.1371/journal.pone.0142081)
Supplement: S3 Table — (DOCX) [file pone.0142081.s006.docx]

SUPPLEMENTAL DATA:

**S3 Table**. **Top Differentially Expressed Genes**

| Probe | Symbol | Description | Treatment Effect ( Post vs Pre) FCH | | |
| --- | --- | --- | --- | --- | --- |
|  |  |  | Responders | Non-Responders | Placebo |
| 204385_at | KYNU | Kynureninase | -23.29 | -1.06 | -1.1 |
| 207602_at | TMPRSS11D | transmembrane protease, serine 11D | -15.51 | 1.4 | -1.21 |
| 205863_at | S100A12 | S100 calcium binding protein A12 | -15.4 | -1.09 | 0 |
| 219795_at | SLC6A14 | solute carrier family 6 (amino acid transporter), member 14 | -14.07 | 1.75 | -1.98 |
| 202988_s_at | RGS1 | regulator of G-protein signaling 1 | -14.02 | 1.12 | 1.09 |
| 205569_at | LAMP3 | lysosomal-associated membrane protein 3 | -13.47 | 1.28 | 1.19 |
| 211906_s_at | SERPINB4 | serpin peptidase inhibitor, clade B (ovalbumin), member 4 | -12.89 | 1.82 | -1.34 |
| 232074_at | PRSS27 | protease, serine 27 | -12.83 | 1.08 | -2.17 |
| 202376_at | SERPINA3 | serpin peptidase inhibitor, clade A (alpha-1 antiproteinase, antitrypsin), member 3 | -11.82 | 1.11 | -3.78 |
| 209921_at | SLC7A11 | solute carrier family 7 (anionic amino acid transporter light chain, xc- system), member 11 | -11.7 | -1.05 | 1.06 |
| 220322_at | IL36G | interleukin 36, gamma | -11.24 | 1.11 | -1.2 |
| 220664_at | SPRR2C | small proline-rich protein 2C (pseudogene) | -10.07 | -1.01 | -1.89 |
| 219403_s_at | HPSE | Heparanase | -8.57 | -1.01 | -1.52 |
| 36711_at | MAFF | v-maf musculoaponeurotic fibrosarcoma oncogene homolog F (avian) | -7.9 | 1.22 | -1.31 |
| 203148_s_at | TRIM14 | tripartite motif containing 14 | -7.31 | 1.66 | 1.2 |
| 226702_at | CMPK2 | cytidine monophosphate (UMP-CMP) kinase 2, mitochondrial | -7.18 | 1.38 | 1.02 |
| 216714_at | CCL13 | chemokine (C-C motif) ligand 13 | -7.15 | -1.19 | -1.71 |
| 231779_at | IRAK2 | interleukin-1 receptor-associated kinase 2 | -6.58 | 1.76 | -1.42 |
| 220066_at | NOD2 | nucleotide-binding oligomerization domain containing 2 | -5.17 | 1.11 | -1.16 |
| 207861_at | CCL22 | chemokine (C-C motif) ligand 22 | -5.12 | 1.23 | 1.32 |
| 235276_at | EPSTI1 | epithelial stromal interaction 1 (breast) | -4.94 | 1.06 | -1.61 |
| 203124_s_at | SLC11A2 | solute carrier family 11 (proton-coupled divalent metal ion transporters), member 2 | -4.93 | 1.27 | -1.07 |
| 226811_at | FAM46C | family with sequence similarity 46, member C | -4.79 | 1.39 | 1.08 |
| 209719_x_at | SERPINB3 | serpin peptidase inhibitor, clade B (ovalbumin), member 3 | -4.56 | -1.2 | -1.13 |
| 205014_at | FGFBP1 | fibroblast growth factor binding protein 1 | -4.34 | 1.73 | -1.14 |
| 222500_at | PPIL1 | peptidylprolyl isomerase (cyclophilin)-like 1 | -4.21 | 1.07 | -1.13 |
| 1553103_at | NFX1 | nuclear transcription factor, X-box binding 1 | -3.91 | -1.13 | -1.14 |
| 210813_s_at | XRCC4 | X-ray repair complementing defective repair in Chinese hamster cells 4 | -3.88 | 1.18 | 1.09 |
| 215051_x_at | AIF1 | allograft inflammatory factor 1 | -3.77 | 1.03 | 1.02 |
| 213174_at | TTC9 | tetratricopeptide repeat domain 9 | -3.5 | -1.28 | -1.3 |
| 235232_at | GMEB1 | glucocorticoid modulatory element binding protein 1 | -3.39 | -1.09 | -1.1 |
| 225788_at | RRP36 | ribosomal RNA processing 36 homolog (S. cerevisiae) | -3.36 | -1.05 | -1.48 |
| 210260_s_at | TNFAIP8 | tumor necrosis factor, alpha-induced protein 8 | -3.33 | 1.23 | -1.11 |
| 227184_at | PTAFR | platelet-activating factor receptor | -3.3 | 1.15 | -1.36 |
| 219037_at | RRP15 | ribosomal RNA processing 15 homolog (S. cerevisiae) | -3.2 | 1.06 | -1.33 |
| 225283_at | ARRDC4 | arrestin domain containing 4 | -3.19 | 1.24 | -1.08 |
| 216379_x_at | CD24 | CD24 molecule | -3.17 | -1.07 | -1.18 |
| 213131_at | OLFM1 | olfactomedin 1 | -3.17 | 1.45 | -1.18 |
| 228359_at | UBASH3B | ubiquitin associated and SH3 domain containing B | -3.12 | -1.09 | -1.34 |
| 1557458_s_at | SHB | Src homology 2 domain containing adaptor protein B | -3.11 | -1.16 | -1.23 |
| 242020_s_at | ZBP1 | Z-DNA binding protein 1 | -3.08 | -1.24 | -1.1 |
| 209762_x_at | SP110 | SP110 nuclear body protein | -3.08 | -1.25 | -1.2 |
| 204679_at | KCNK1 | potassium channel, subfamily K, member 1 | -3.06 | 1.32 | -1.24 |
| 211366_x_at | CASP1 | caspase 1, apoptosis-related cysteine peptidase | -3.05 | 1.08 | 1.08 |
| 217835_x_at | C20orf24 | chromosome 20 open reading frame 24 | -2.89 | 1.26 | -1.07 |
| 209734_at | NCKAP1L | NCK-associated protein 1-like | -2.88 | -1.07 | -1.09 |
| 207356_at | DEFB4A | defensin, beta 4A | -2.88 | 1.18 | -1.09 |
| 215617_at | SPATS2L | spermatogenesis associated, serine-rich 2-like | -2.85 | 1 | -1.2 |
| 214512_s_at | SUB1 | SUB1 homolog (S. cerevisiae) | -2.85 | 1.5 | -1 |
| 218239_s_at | GTPBP4 | GTP binding protein 4 | -2.84 | 1.21 | -1.16 |
| 218269_at | DROSHA | drosha, ribonuclease type III | -2.82 | -1.41 | -1.19 |
| 223299_at | SEC11C | SEC11 homolog C (S. cerevisiae) | -2.19 | 1.17 | -1.11 |
| 225315_at | MRPL21 | mitochondrial ribosomal protein L21 | -2.19 | 1.38 | -1.06 |
| 213857_s_at | CD47 | CD47 molecule | -2.15 | -1.05 | -1.08 |
| 209922_at | BRAP | BRCA1 associated protein | -2.11 | 1.01 | -1.1 |
