# Supplementary material for: A Randomized, Placebo-Controlled Study of SRT2104, a SIRT1 Activator, in Patients with Moderate to Severe Psoriasis
Source: PLoS One. 2015 Nov 10;10(11):e0142081. doi: 10.1371/journal.pone.0142081 (PMC4640558; doi:10.1371/journal.pone.0142081)
Supplement: S4 Table — (DOCX) [file pone.0142081.s007.docx]

SUPPLEMENTAL DATA:

**S4 Table**. **GSEA Analysis of the Responder Group**
